# Supplementary material for: Type I Interferon Signaling Disrupts the Hepatic Urea Cycle and Alters Systemic Metabolism to Suppress T Cell Function
Source: Immunity. 2019 Dec 17;51(6):1074–1087.e9. doi: 10.1016/j.immuni.2019.10.014 (PMC6926485; doi:10.1016/j.immuni.2019.10.014)
Supplement: Document S1. Figures S1–S6 [file mmc1.pdf]

Immunity, Volume *51*

Supplemental Information

Type I Interferon Signaling Disrupts the Hepatic Urea Cycle and Alters Systemic Metabolism to Suppress T Cell Function

Alexander Lercher, Anannya Bhattacharya, Alexandra M. Popa, Michael Caldera, Moritz F. Schlapansky, Hatoon Baazim, Benedikt Agerer, Bettina Gürtl, Lindsay Kosack, Peter Májek, Julia S. Brunner, Dijana Vitko, Theresa Pinter, Jakob-Wendelin Genger, Anna Orlova, Natalia Pikor, Daniela Reil, Maria Ozsvár-Kozma, Ulrich Kalinke, Burkhard Ludewig, Richard Moriggl, Keiryn L. Bennett, Jörg Menche, Paul N. Cheng, Gernot Schabbauer, Michael Trauner, Kristaps Klavins, and Andreas Bergthaler

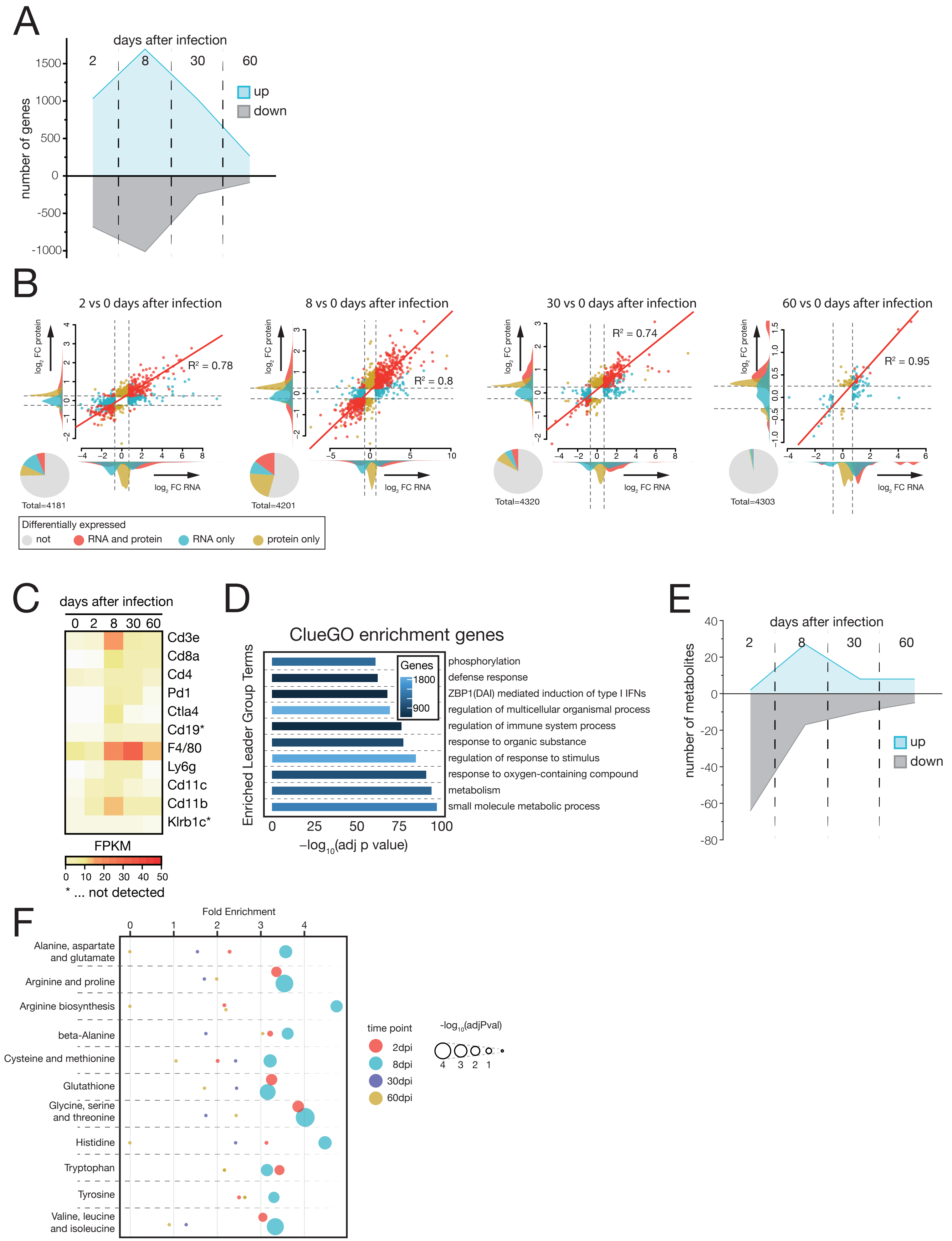
A
days after infection
2
8
30
60
up
down
number of genes
1500
1000
500
0
-500
-1000
B
2 vs 0 days after infection
8 vs 0 days after infection
30 vs 0 days after infection
60 vs 0 days after infection
log2 FC protein
log2 FC RNA
R² = 0.78
R² = 0.8
R² = 0.74
R² = 0.95
Total=4181
Total=4201
Total=4320
Total=4303
Differentially expressed
not
RNA and protein
RNA only
protein only
C
days after infection
0 2 8 30 60
Cd3e
Cd8a
Cd4
Pd1
Ctla4
Cd19*
F4/80
Ly6g
Cd11c
Cd11b
Klrb1c*
FPKM
0 10 20 30 40 50
* ... not detected
D
ClueGO enrichment genes
Enriched Leader Group Terms
Genes
1800
900
phosphorylation
defense response
ZBP1(DAI) mediated induction of type I IFNs
regulation of multicellular organismal process
regulation of immune system process
response to organic substance
regulation of response to stimulus
response to oxygen-containing compound
metabolism
small molecule metabolic process
0 25 50 75 100
–log10(adj p value)
E
days after infection
2
8
30
60
number of metabolites
40
20
0
-20
-40
-60
-80
up
down
F
Fold Enrichment
0 1 2 3 4
Alanine, aspartate and glutamate
Arginine and proline
Arginine biosynthesis
beta-Alanine
Cysteine and methionine
Glutathione
Glycine, serine and threonine
Histidine
Tryptophan
Tyrosine
Valine, leucine and isoleucine
time point
2dpi
8dpi
30dpi
60dpi
-log10(adjPval)
4 3 2 1

Figure S1. Systems biology approach to transcriptomic, proteomic and metabolomic changes in liver tissue and systemic metabolism during LCMV Cl13 infection. Related to Figure 1.

(A) Number of significantly up- or downregulated genes in LCMV-infected liver tissue 2, 8, 30 or 60 days after infection compared to naïve liver tissue (n = 3). (B) Correlation of transcriptomic and proteomic changes at the corresponding time points after LCVM infection (n = 3). (C) Heatmap of expression values (FPKM) of indicated immune cell markers in liver tissue during the course of LCMV infection (n = 3). (D) Enriched GO terms and pathways (ClueGO) on the union of significantly differentially expressed transcripts at any time point (n = 3). (E) Number of significantly up- or downregulated serum metabolites of LCMV-infected wild type mice compared to naïve serum metabolite levels (n = 4). (F) Enrichment analyses of significantly regulated transcripts for amino acid metabolic pathways (KEGG) at the indicated time points after LCMV infection (n = 3). For (A-F) transcriptomic and proteomic data is derived from one experiment.

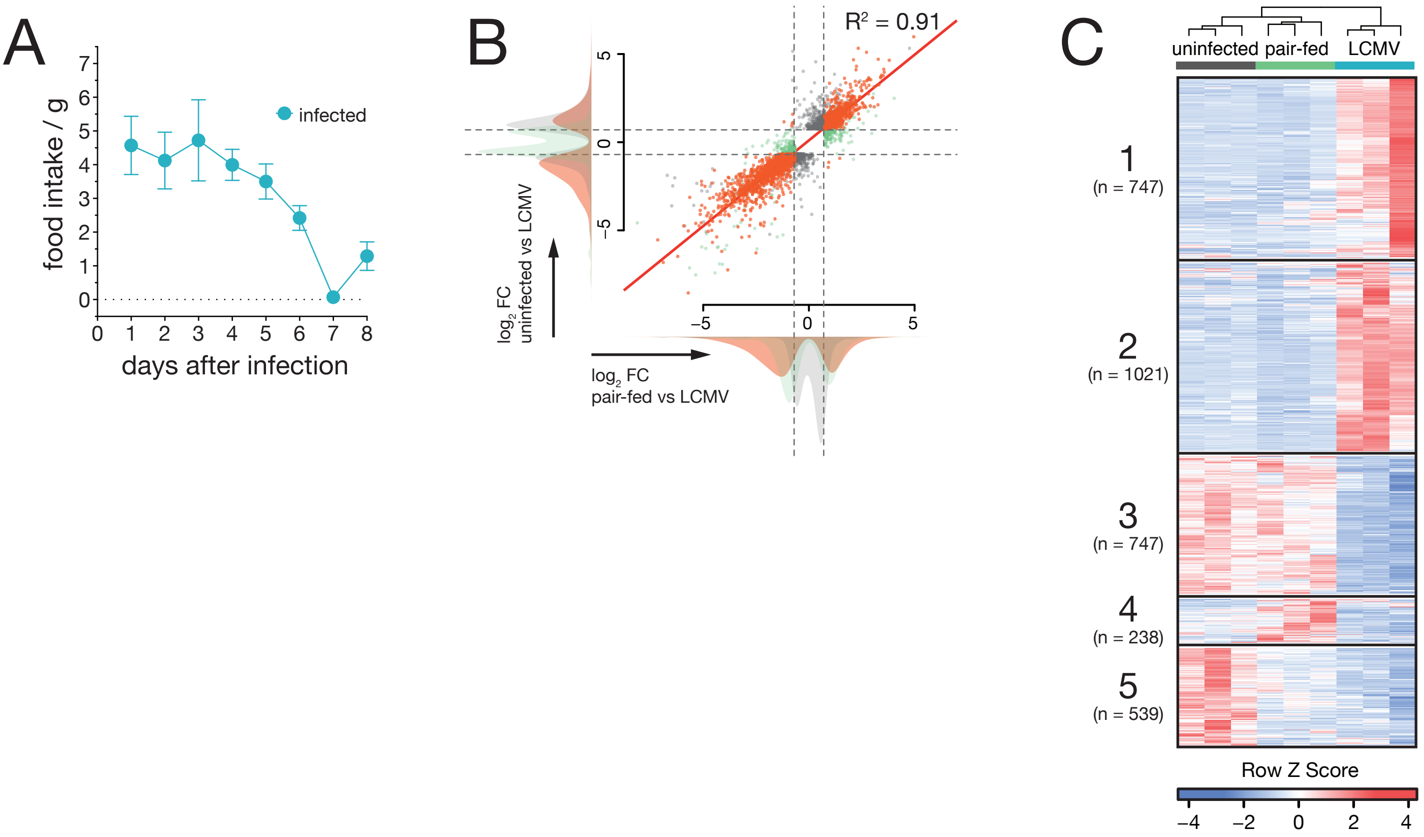

Figure S2. Transcriptome analyses of naïve and pair-fed compared to LCMV-infected animals. Related to Figure 2.

(A) Food intake of LCMV-infected animals (n = 3) up to 8 days post infection. (B) Correlation of significantly regulated transcripts in the livers of naïve and pair-fed animals (8 days) compared to LCMV-infected animals (n = 3). (C) Hierarchical clustering (FPKM, k-means, Pearson's correlation) of significantly deregulated transcripts in liver tissue of naïve, pair-fed and LCMV-infected animals (n = 3). For (A-C) transcriptomic data is derived from one experiment. Symbols represent the arithmetic mean ±S.E.M.

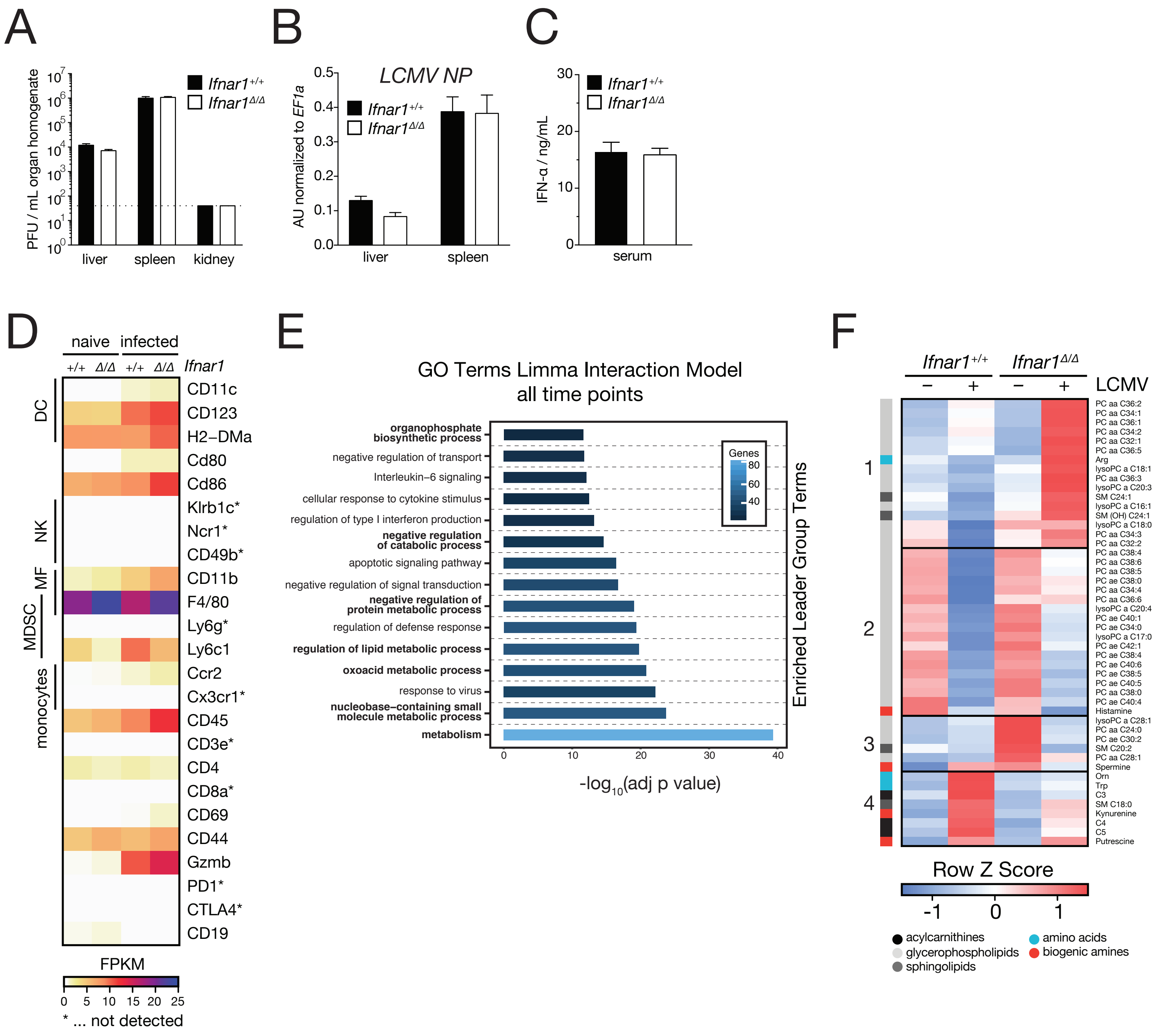

Figure S3. Transcriptome and systemic metabolome analyses of LCMV Cl13 infected *Alb-Cre ERT2 Ifnar1*$^{fl/fl}$ mice. Related to Figure 3.

(A) Viremia and (B) RNemia in organs (n = 4), and (C) IFN-α serum levels of *Alb-Cre ERT2 Ifnar1*$^{fl/fl}$ (*Ifnar1*$^{Δ/Δ}$) and *Ifnar1*$^{+/+}$ mice 1.5 days after infection (n = 4). (D) Heatmap of expression values (FPKM) of the indicated immune cell markers in naïve or infected *Ifnar1*$^{+/+}$ and *Ifnar1*$^{Δ/Δ}$ mice (n = 3). (E) Enriched GO terms on the union of significantly regulated (limma interaction model) genes (n = 3). (F) Significantly regulated serum metabolites in naïve and infected *Ifnar1*$^{Δ/Δ}$ and *Ifnar1*$^{+/+}$ animals (n = 3, k-means, Pearson's correlation). For (A-C) one of two representative experiments are shown. For (D-F) transcriptomic and metabolomic data is derived from one experiment. AU = arbitrary units. Symbols represent the arithmetic mean ±S.E.M. Dotted line implicates limit of detection. ns = not significant * $P < 0.05$ ** $P < 0.01$ *** $P < 0.001$(Student's t-test).

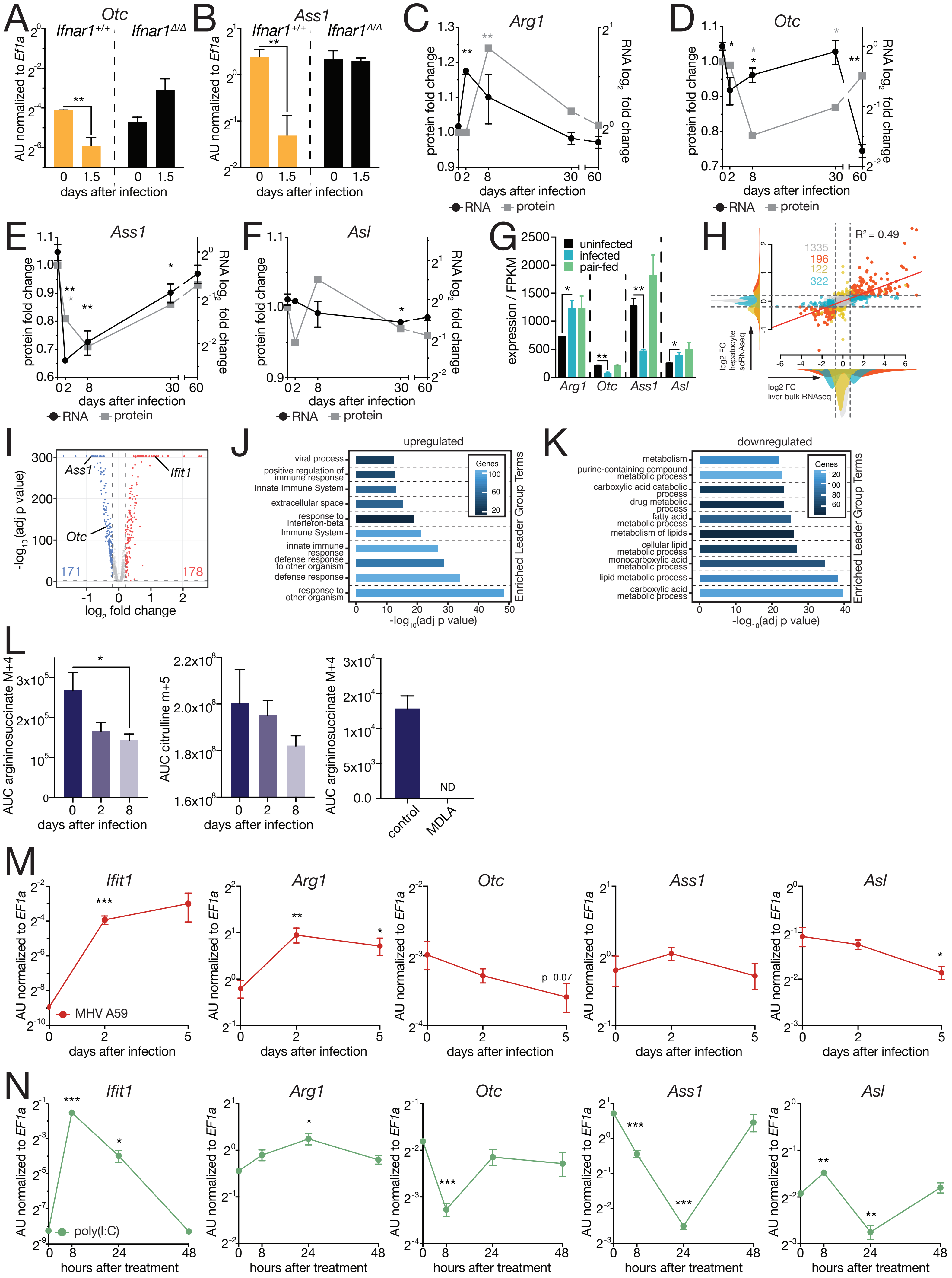

A
Otc
Ifnar1+/+
Ifnar1Δ/Δ
AU normalized to Ef1a
days after infection
B
Ass1
C
Arg1
protein fold change
RNA log2 fold change
RNA
protein
D
Otc
E
Ass1
F
Asl
G
uninfected
infected
pair-fed
expression / FPKM
H
R2 = 0.49
1335
196
122
322
log2 FC hepatocyte scRNAseq
log2 FC liver bulk RNAseq
I
Ass1
Ifit1
Otc
171
178
-log10(adj p value)
log2 fold change
J
upregulated
viral process
positive regulation of immune response
Innate Immune System
extracellular space
response to interferon-beta
Immune System
innate immune response
defense response to other organism
defense response
response to other organism
Genes
Enriched Leader Group Terms
-log10(adj p value)
K
downregulated
metabolism
purine-containing compound metabolic process
carboxylic acid catabolic process
drug metabolic process
fatty acid metabolic process
metabolism of lipids
cellular lipid metabolic process
monocarboxylic acid metabolic process
lipid metabolic process
carboxylic acid metabolic process
L
AUC argininosuccinate M+4
AUC citrulline m+5
ND
control
MDLA
M
Ifit1
Arg1
Otc
Ass1
Asl
MHV A59
p=0.07
N
poly(I:C)
hours after treatment

Figure S4. IFNAR1 signaling and viral infection perturb the urea cycle in hepatocytes. Related to Figure 4.

Real-time PCR of *Otc* (A) and *Ass1* (B) in *Alb-Cre ERT2 Ifnar1fl/fl* (*Ifnar1*$^{\Delta/\Delta}$) and *Ifnar1*$^{+/+}$ mice 1.5 days after infection (n = 3-4). (D-F) Long-term kinetics of urea cycle gene transcript and protein abundances of *Arg1*, *Otc*, *Ass1* and *Asl* in liver tissue upon LCMV-infection (n = 3). (G) Expression values (FPKM) of *Arg1*, *Otc*, *Ass1* and *Asl* of naïve, pair-fed and LCMV-infected (8 days after infection) wild type animals (n = 3). (H) Correlation plot (Spearman correlation) of significantly changed genes in scRNA-seq dataset (n = 2, pooled for each condition) compared to RNA-seq data obtained from bulk liver tissue (n = 3, Figure 1). (I) Volcano plot of scRNA-seq data displaying up- and downregulated transcripts (n = 2, pooled for each condition); arrows indicate the urea cycle genes *Otc* and *Ass1* and the type I interferon stimulated gene *Ifit1*. (J, K) Enriched GO terms of significantly up- and downregulated genes in hepatocytes derived from naïve or LCMV-infected mice (2 days, n = 2, pooled for each condition). (L) Detection of ^{13}C labeled reaction products for ASS1 ($^{13}C_4$ argininosuccinate) and OTC ($^{13}C_5$ citrulline) after pulsing liver lysates of naïve or LCMV-infected animals (2 and 8 days after infection) for 30 minutes with the respective ^{13}C labeled substrates for OTC and ASS1 (n = 4-5) and detection of the ASS1 reaction product $^{13}C_4$ argininosuccinate in naïve liver lysates in the presence or absence of the ASS1 inhibitor α-methyl-DL-aspartic acid (n = 3). (M) Expression of *Ifit1* and urea cycle-associated genes in liver tissue of mice infected with murine hepatitis virus (MHV) strain A59 measured by real-time PCR (n = 5). (N) Expression of *Ifit1* and urea cycle-associated genes in liver tissue upon poly(I:C) treatment of wild type mice measured by real-time PCR (n = 3). For (A-B) one of two representative experiments are shown. For (D-N) data is derived from one experiment. AU = arbitrary units, AUC = area under curve. Symbols represent the arithmetic mean ±S.E.M. ns = not significant * $P < 0.05$ ** $P < 0.01$ (Student's t-test).

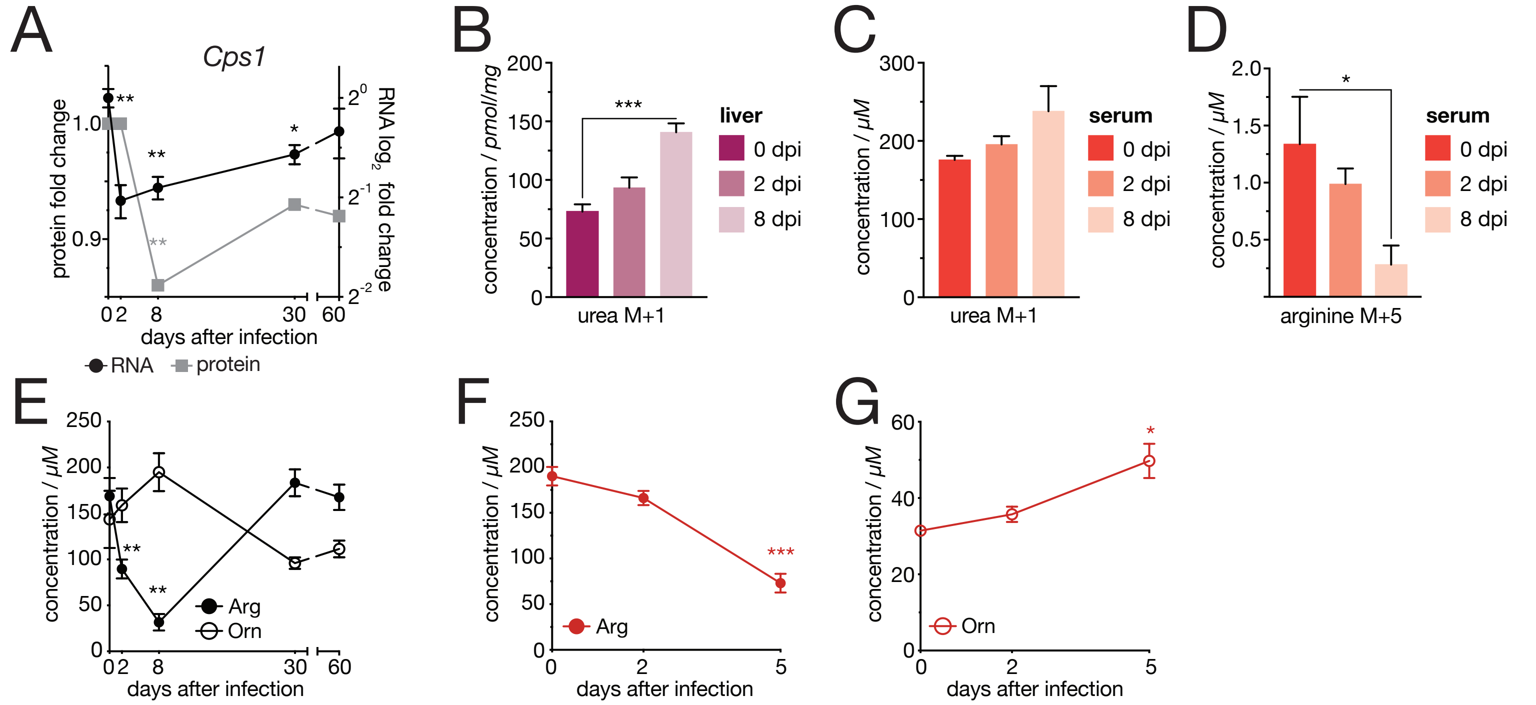

Figure S5. Viral infection stalls the hepatic urea cycle and correlates with changes in serum levels of arginine and ornithine. Related to Figure 5.

(A) Transcript and protein levels of *Cps1* in liver tissue of mice infected with LCMV up to 60 days after infection (n = 3). Concentrations of $^{13}C_1$ labeled urea in (B) liver tissue, (C) serum and (D) $^{13}C_5$ labelled arginine in serum of naïve and LCMV-infected (2 and 8 days after infection) wild type mice (n= 3-6). (E) Serum levels of arginine and ornithine in LCMV-infected wild type mice up to 60 days after infection. (F, G) Serum levels of arginine and ornithine in mice infected with murine hepatitis virus (MHV) strain A59 up to 5 days after infection (n = 5). For (E) two individual biological experiments were pooled. For (A–D and F-G) transcriptomic, metabolomic and metabolite tracing data is derived from one experiment. Symbols represent the arithmetic mean ±S.E.M. ns = not significant * $P < 0.05$ ** $P < 0.01$ (Student's t-test).

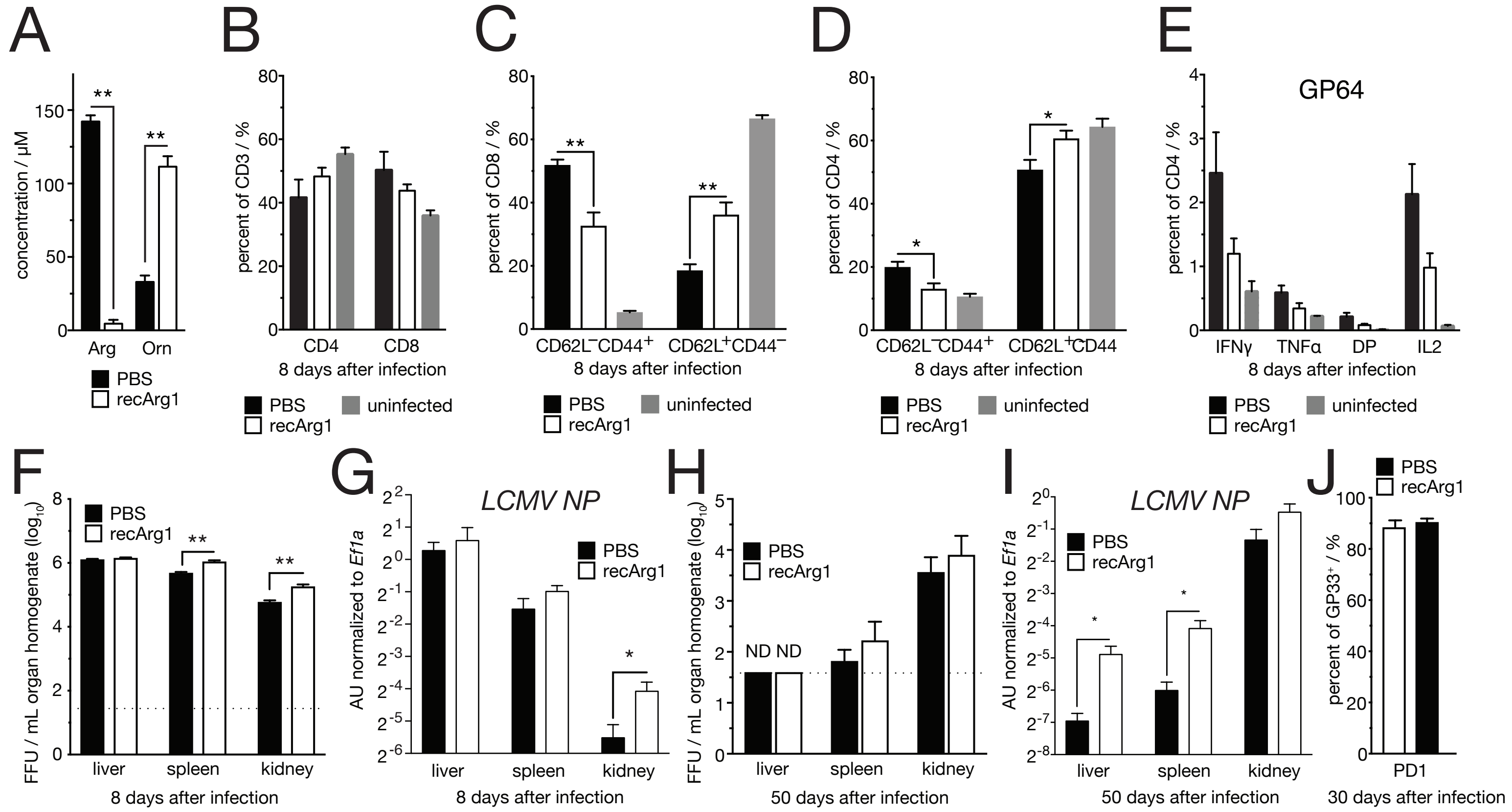

Figure S6. Arginase1 treatment does not affect splenic CD4 and CD8 T cell abundances and mildly impairs antiviral CD4 T cell responses and affects organ viral load. Related to Figure 6.

(A) Serum arginine and ornithine levels in wild type mice (n = 4) 2 days after treatment with pegylated recombinant human Arginase 1 (recArg1, BCT-100). (B) Percentage of $CD4^+$ and $CD8^+$ cells of $CD3^+$ splenocytes at 8 days after LCMV clone 13 infection and recArg1 treatment (n = 5). (C) $CD62L^-CD44^+$ (effector) and $CD62L^+CD44^-$ (naïve) CD8 and (D) CD4 splenic T cells (n = 5). (E) IFNγ, TNFα and IL-2 production of LCMV GP64-specific splenic CD4 T cells 8 days after infection. (F) Viraemia and (G) RNemia of liver, spleen and kidney 8 days after infection (n = 5). (H) Viraemia and (I) RNemia of liver, spleen and kidney 50 days after infection (n = 4-5). (J) PD1 expression on GP33-specific CD8 T cells in blood 30 days after infection (n = 5). For (B-I) one of at least two representative experiments are shown. For (A) data is derived from one experiment. AU = arbitrary units. Symbols represent the arithmetic mean ±S.E.M. n = 4-5. ns = not significant. ND = not detected. Dotted line implicates limit of detection. * $P < 0.05$ ** $P < 0.01$ (Student's t-test).

Table S1: Transcriptomic and proteomic data of LCMV-infected wild type mice. Related to Figure 1.

Table S2: Enrichment cluster analysis of differentially expressed genes. Related to Figure 1.

Table S3: Metabolomics data of LCMV-infected mice. Related to Figure 1.

Table S4: Transcriptomic and gene ontology enrichment data of pair-feeding experiment. Related to Figure 2.

Table S5: Transcriptomic and gene ontology enrichment data of *Alb-Cre ERT2 Ifnar1* mice. Related to Figure 3.
