# Supplementary material for: Type I Interferon Signaling Disrupts the Hepatic Urea Cycle and Alters Systemic Metabolism to Suppress T Cell Function
Source: Immunity. 2019 Dec 17;51(6):1074–1087.e9. doi: 10.1016/j.immuni.2019.10.014 (PMC6926485; doi:10.1016/j.immuni.2019.10.014)
Supplement: Document S2. Article plus Supplemental Information [file mmc7.pdf]

Article

Immunity

Type I Interferon Signaling Disrupts the Hepatic Urea Cycle and Alters Systemic Metabolism to Suppress T Cell Function

Graphical Abstract

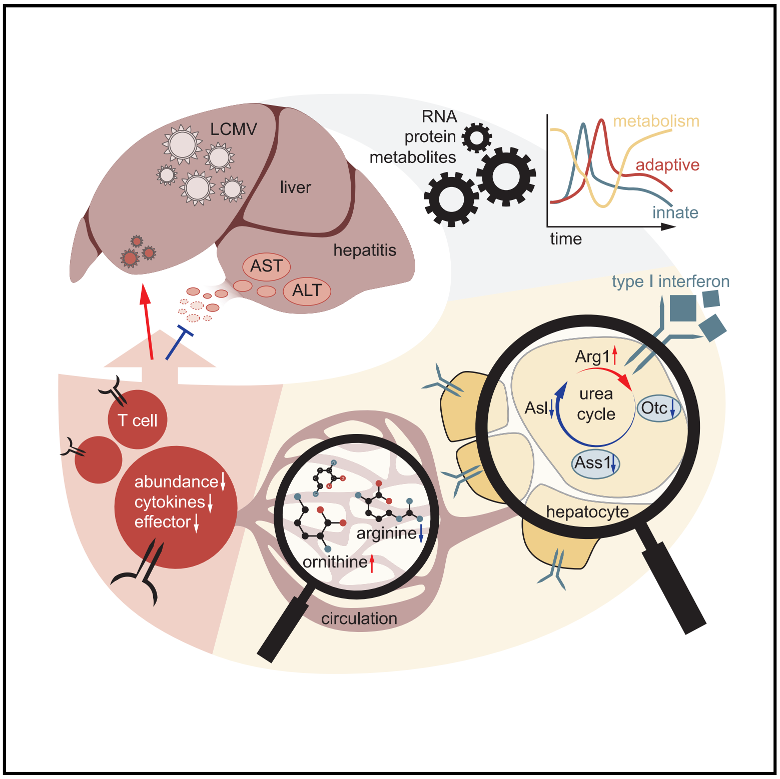

Highlights

- Chronic viral infection causes long-term transcriptome and proteome changes in the liver
- Hepatocyte-intrinsic type I interferon (IFN-I) signaling regulates hepatic metabolism
- IFN-I signaling reprograms the urea cycle in hepatocytes and alters serum metabolites
- Serum levels of arginine and ornithine modulate T cell responses and pathology

Authors

Alexander Lercher,
Anannya Bhattacharya,
Alexandra M. Popa, ...,
Michael Trauner, Kristaps Klavins,
Andreas Bergthaler

Correspondence

abergthaler@cemm.oeaw.ac.at

In Brief

The liver is a central regulator of metabolism, but how infection-induced changes in liver metabolism affect immune responses and pathology remains enigmatic. Lercher et al. demonstrate that hepatocyte-intrinsic type I interferon (IFN-I) represses metabolism during chronic viral infection. Specifically, IFN-I disrupts the urea cycle in hepatocytes, altering serum arginine and ornithine levels, which dampen antiviral T cells responses and liver pathology.

Lercher et al., 2019, Immunity *51*, 1074–1087
December 17, 2019 © 2019 The Author(s). Published by Elsevier Inc.
https://doi.org/10.1016/j.immuni.2019.10.014

CellPress
Immunity
Article

Type I Interferon Signaling Disrupts the Hepatic Urea Cycle and Alters Systemic Metabolism to Suppress T Cell Function

Alexander Lercher,[1,14] Anannya Bhattacharya,[1,13,14] Alexandra M. Popa,[1] Michael Caldera,[1] Moritz F. Schlapansky,[1] Hatoon Baazim,[1] Benedikt Agerer,[1] Bettina Gürtl,[1] Lindsay Kosack,[1] Peter Májek,[1] Julia S. Brunner,[2,3] Dijana Vitko,[1,4] Theresa Pinter,[1,5] Jakob-Wendelin Genger,[1] Anna Orlova,[6] Natalia Pikor,[7] Daniela Reil,[1] Maria Ozsvár-Kozma,[1,8] Ulrich Kalinke,[9] Burkhard Ludewig,[7] Richard Moriggl,[6,10] Keiryn L. Bennett,[1] Jörg Menche,[1] Paul N. Cheng,[11] Gernot Schabbauer,[2,3] Michael Trauner,[12] Kristaps Klavins,[1] and Andreas Bergthaler[1,15,*]

[1]CeMM Research Center for Molecular Medicine or the Austrian Academy of Sciences, Lazarettgasse 14 AKH BT25.3, 1090 Vienna, Austria
[2]Department of Thrombosis Research and Vascular Biology, Medical University of Vienna, 1090 Vienna, Austria
[3]Christian Doppler Laboratory for Arginine Metabolism in Rheumatoid Arthritis and Multiple Sclerosis, 1090 Vienna, Austria
[4]Department of Urology, Boston Children's Hospital, Harvard Medical School, Boston, MA 02115, USA
[5]Research Institute of Molecular Pathology (IMP), Vienna BioCenter (VBC), 1030 Vienna, Austria
[6]Institute of Animal Breeding and Genetics, University of Veterinary Medicine Vienna, 1210 Vienna, Austria
[7]Institute of Immunobiology, Kantonsspital St. Gallen, 9007 St. Gallen, Switzerland
[8]Department for Laboratory Medicine, Medical University of Vienna, 1090 Vienna, Austria
[9]Institute for Experimental Infection Research, TWINCORE, Centre for Experimental and Clinical Infection Research, a joint venture between the Helmholtz Centre for Infection Research, Braunschweig, and the Hannover Medical School, 30625 Hannover, Germany
[10]Medical University of Vienna, 1090 Vienna, Austria
[11]Bio-Cancer Treatment International Limited, Hong Kong, China
[12]Division of Gastroenterology & Hepatology, Department of Internal Medicine III, Medical University of Vienna, 1090 Vienna, Austria
[13]Present address: Merck Research Laboratories, 33 Avenue Louis Pasteur, Boston, MA 02115, USA
[14]These authors contributed equally
[15]Lead Contact
*Correspondence: abergthaler@cemm.oeaw.ac.at
https://doi.org/10.1016/j.immuni.2019.10.014

SUMMARY

Infections induce complex host responses linked to antiviral defense, inflammation, and tissue damage and repair. We hypothesized that the liver, as a central metabolic hub, may orchestrate systemic metabolic changes during infection. We infected mice with chronic lymphocytic choriomeningitis virus (LCMV), performed RNA sequencing and proteomics of liver tissue, and integrated these data with serum metabolomics at different infection phases. Widespread reprogramming of liver metabolism occurred early after infection, correlating with type I interferon (IFN-I) responses. Viral infection induced metabolic alterations of the liver that depended on the interferon alpha/beta receptor (IFNAR1). Hepatocyte-intrinsic IFNAR1 repressed the transcription of metabolic genes, including *Otc* and *Ass1*, which encode urea cycle enzymes. This led to decreased arginine and increased ornithine concentrations in the circulation, resulting in suppressed virus-specific CD8$^+$ T cell responses and ameliorated liver pathology. These findings establish IFN-I-induced modulation of hepatic metabolism and the urea cycle as an endogenous mechanism of immunoregulation.

INTRODUCTION

Pathogens and concurrent tissue damage elicit complex context-dependent inflammatory response programs (Hotamisligil, 2017). Chronic infections represent a particular challenge for the host organism, which is exposed to prolonged inflammation that may predispose to various co-morbidities, such as susceptibility to secondary infections and cancer (Okin and Medzhitov, 2012; Rehermann and Nascimbeni, 2005; Virgin et al., 2009). The same inflammatory pathways can also control cellular tissue homeostasis and metabolism (Kotas and Medzhitov, 2015; Medzhitov, 2008; O'Neill and Pearce, 2016). Different cell types and organs communicate with each other through soluble cytokines, thereby determining the quality, magnitude, and duration of both local and systemic immune responses (Medzhitov, 2008). The liver is a central metabolic organ but also represents an immunoregulatory hub between blood-borne pathogens and the immune system (Jenne and Kubes, 2013; Protzer et al., 2012; Racanelli and Rehermann, 2006). Hepatocytes are the functional unit of the liver parenchyma and the most abundant cell type of the liver. As such, their principal task is the turnover of metabolites during homeostasis. Yet they are also important immune signaling platforms that produce and react to a range of cytokines upon inflammation (Crispe, 2016; Racanelli and Rehermann, 2006; Zhou et al., 2016). Hepatocytes themselves are permissible to a variety of chronic viruses, including hepatitis B virus (HBV) and hepatitis C virus (HCV) in humans and lymphocytic choriomeningitis virus (LCMV) in

 © 2019 The Author(s). Published by Elsevier Inc.
This is an open access article under the CC BY license (http://creativecommons.org/licenses/by/4.0/).

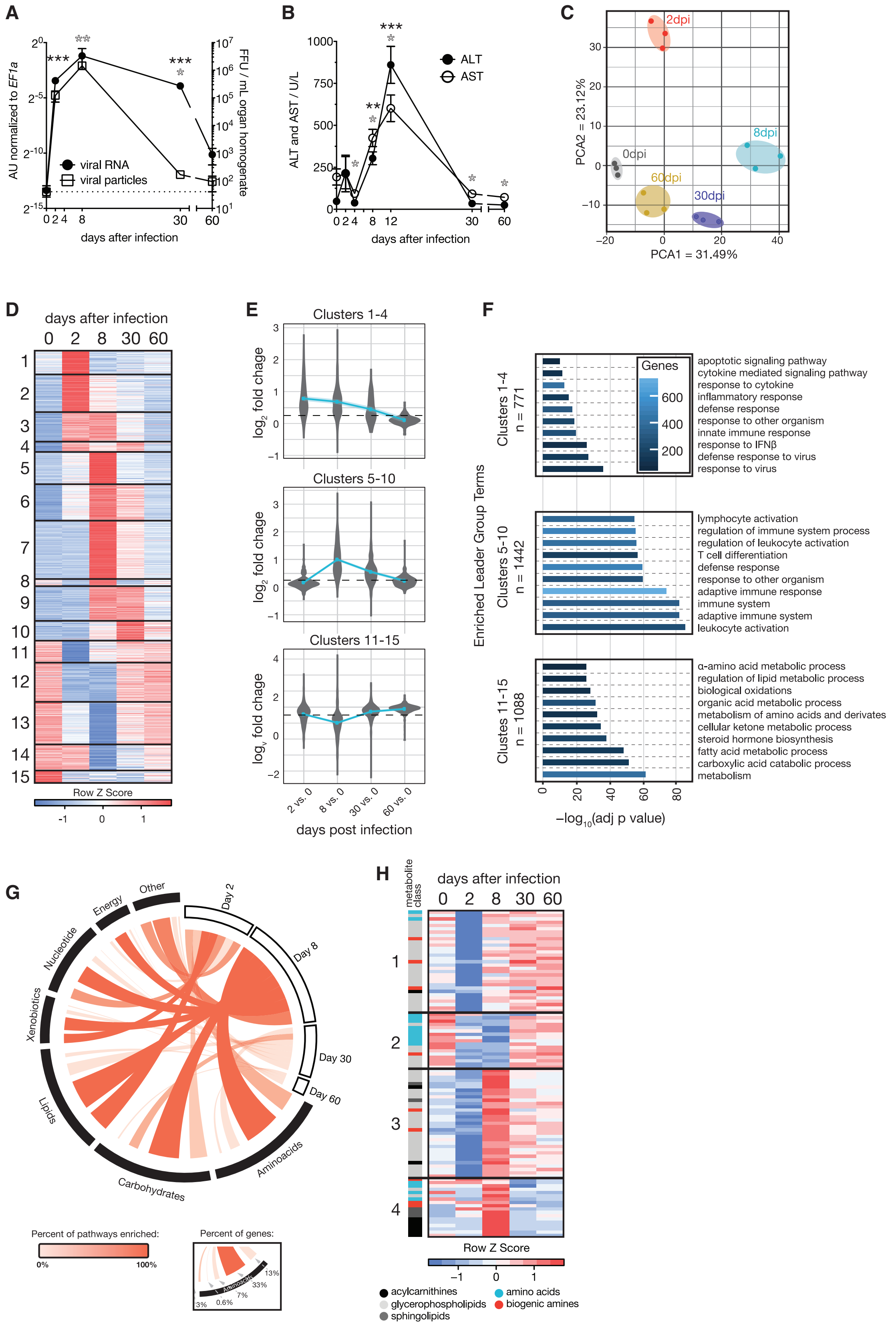

(legend on next page)

mice (Guidotti et al., 1999; Rehermann and Nascimbeni, 2005). The chronic infection model of LCMV represents a well-established and pathophysiologically relevant experimental model for the study of host-pathogen interactions and immune responses that induce a vigorous CD8 T-cell-dependent hepatitis (Zehn and Wherry, 2015; Zinkernagel et al., 1986). The involved immunopathologic mechanisms seen in the LCMV model are similar to those observed in patients chronically infected with HBV or HCV (Guidotti et al., 1999; Rehermann and Nascimbeni, 2005).

Soluble inflammatory signals act mainly through cytokine receptors (Hotamisligil, 2017; Protzer et al., 2012; Racanelli and Rehermann, 2006). Type I interferons (IFN-Is) are central antiviral cytokines that signal through the ubiquitously expressed IFNAR receptor, which is composed of the two subunits IFNAR1 and IFNAR2. This induces the expression of a broad array of genes described as interferon-stimulated genes (ISGs). ISGs exert antiviral functions by direct interference with viral replication and immunoregulatory properties (McNab et al., 2015; Schoggins et al., 2011). More recently, IFN-Is are also recognized as modulators of metabolism, such as cellular lipid metabolism and redox homeostasis (Bhattacharya et al., 2015; Pantel et al., 2014; Wu et al., 2016; York et al., 2015). Cytokine-induced regulation of liver metabolism is expected to result in altered metabolite turnover and release that impacts distal organs (van den Berghe, 1991; Norata et al., 2015). Immune cells and in particular T cells critically depend on certain metabolites to efficiently perform their functions and are thus susceptible to altered metabolite availability (Chang et al., 2015; Geiger et al., 2016; Johnson et al., 2018; Pearce et al., 2009). In line with this, a frequent immune evasion mechanism of cancer is the depletion of essential amino acids or glucose in the tumor microenvironment (Buck et al., 2017; Chang et al., 2015; Ma et al., 2017; Murray, 2016).

In this study, we investigated the chronic infection model of LCMV using an unbiased integrative approach to unveil inflammation-driven endogenous regulation of liver metabolism and its impact on systemic immune responses and tissue pathology.

RESULTS

Identification of Inflammatory-Metabolic Changes in the Liver during Chronic Infection

We infected C57BL/6J wild-type mice with 2×10^6 focus forming units (FFUs) of the chronic strain clone 13 of LCMV and quantified infectious virus particles and viral RNA in the liver (Figure 1A). This confirmed the peak of viral propagation around day 8 after infection with a subsequent decline of viral loads. Liver damage was assessed by the clinical hallmark parameters alanine aminotransferase (ALT) and aspartate aminotransferase (AST) that peaked on day 8–12 after infection (Figure 1B). In an unbiased approach to virus-induced changes in the liver, we collected liver tissue from infected mice at different phases of infection (day 2 ≈ innate phase; day 8 ≈ peak of disease; day 30 ≈ chronic phase; day 60 ≈ resolving phase) and performed transcriptome analyses by RNA sequencing (RNA-seq). The transcriptomic data showed high intra-replica reproducibility and individual samples clustered according to the time course of infection (Figure 1C), highlighting phase-specific changes in gene expression and the gradual recovery of mice by 60 days after infection. In total, 3,626 transcripts were differentially regulated (Table S1A) and the most differentially expressed genes were found on days 2 and 8 after infection (Figure S1A). Hierarchical clustering identified three broad categories of gene expression programs, including transcripts that were found already regulated on day 2 (clusters 1–4) or induced on day 8 (clusters 5–10) as well as transcripts that were repressed during infection (clusters 11–15; Figure 1D; Table S2A). To corroborate transcriptional alterations, we performed tissue proteomics and quantified 5,586 proteins in the liver (Table S1B). Protein changes correlated with differentially expressed transcripts (Figures 1E and S1B; Table S2C). Of the identified clusters, differentially expressed genes associated with antiviral IFN-I signaling were found to be enriched on day 2 (clusters 1–4; Figure 1F; Table S2B) although leukocyte-associated genes were mainly found on day 8 after infection (clusters 5–10; Figure 1F; Table S2B). Some of these differential expression changes are likely to reflect hepatic immune cell infiltration (Figure S1C).

Our enrichment analyses indicated widespread metabolic reprogramming of liver tissue during LCMV infection (Figure S1D), with particularly pronounced effects seen for downregulated metabolic pathways relating to lipid and amino acid metabolism on days 2 and 8 after infection (clusters 11–15; Figure 1F; Table S2B). This is in line with our recent study about the modulation of circulating lipids during acute LCMV infection (Kosack et al., 2017). For an in-depth integration of these metabolic changes, we performed enrichment analyses of the union of differentially regulated transcripts and proteins at each stage of viral infection on Kyoto Encyclopedia of Genes and Genomes (KEGG) metabolic pathways. We identified global reprogramming of processes related to all the major classes of metabolites. Modulation of hepatic pathways involving lipids and essential amino acids was initiated on day 2 and peaking on day 8 after infection

Figure 1. LCMV Cl13 Induces Hepatic Metabolic Reprogramming, Translating to Changes in Systemic Metabolism during the Course of Infection

(A and B) Viremia and RNemia of LCMV-clone-13-infected liver tissue (A; n = 3) and serum alanine transferase (ALT) and aspartate aminotransferase (AST) levels upon LCMV clone 13 infection up to 60 days after infection (B; n = 4–12).

(C) Principal-component analyses (PCA) of liver tissue transcriptomes at 0, 2, 8, 30, and 60 days after infection (n = 3).

(D) Hierarchical clustering (fragments per kilobase of transcript per million [FPKM]; k-means; Pearson's correlation) of significantly changed genes at any indicated time point (n = 3).

(E) Regulation of detected proteins in liver tissue at the corresponding time points (n = 3).

(F) Enriched GO terms and pathways (ClueGO) of transcripts identified in the groups of clusters shown in (D).

(G) Enrichment of the union of significantly regulated metabolic transcripts and proteins on KEGG metabolic database at any time point.

(H) Hierarchical clustering of significantly regulated serum metabolites (n = 4; k-means; Pearson's correlation). dpi, days post-infection.

For (A), (B), and (H), one out of at least two representative experiments is shown. For (C)–(G), transcriptomic and proteomic data are derived from one experiment. Symbols represent the arithmetic mean ± SEM; ns, not significant; *$p < 0.05$; **$p < 0.01$; ***$p < 0.001$ (Student's t test). See also Figure S1.

(Figure 1G; Table S1C). Differentially expressed metabolic processes were also found in the chronic and resolving phases of infection, although the number of modulated transcripts and proteins were lower (Figures 1G, S1A, and S1B).

To address the effects of virus-induced alterations on the metabolic output of the liver, we performed targeted metabolomics of serum, focusing on amino acids, biogenic amines, sphingolipids, acylcarnitines, and glycerophospholipids. We found 99 of 180 metabolites to be significantly regulated at one or more time points (Figure 1H; Table S3A). Downregulation of metabolites on day 2 (Figure S1E) was observed for glycerophospholipids and sphingolipids (Figure 1H; clusters 1 and 3) although acylcarnitines (Figure 1H; cluster 4) were rather upregulated on day 8 after infection (Figure 1H). Systemic amino acids and biogenic amines exhibited a sustained decline from day 2 to day 8 and gradually recovered in the later phases of infection (Figure 1H; cluster 2). This affected almost exclusively essential and semi-essential proteinogenic amino acids (His, Ile, Leu, Met, Trp, Val, and Arg; Figure 1H) and coincided with transcriptional regulation of amino-acid-related metabolic pathways on day 8 (Figures 1F–1H and S1F; Table S1C). Together, our integrated transcriptomic and proteomic analysis linked changes in the liver with altered systemic metabolite levels during chronic viral infection.

Reduced Food Intake during Viral Infection Mildly Affects Gene Expression in the Liver

Mice infected with LCMV strain clone 13 develop infection-associated cachexia (Baazim et al., 2019) and display reduced food intake (Figure S2A). We therefore performed a pair-feeding experiment to investigate the impact of infection-associated changes in food uptake to the metabolic reprogramming of the liver 8 days after infection. Uninfected mice received restricted amounts of food equivalent to the amounts consumed by infected mice. Subsequently, we collected liver tissue of infected, pair-fed, and naive animals and performed transcriptome analyses. These results indicated that most of the observed transcriptional changes were linked to viral infection and independent of anorexic behavior (Figures 2A, S2B, and S2C). Uninfected pair-fed mice showed only minor differential gene expression changes that pertained mainly to lipid metabolism (Figures 2B and 2C; Table S4). Together, these experiments demonstrated that the majority of the observed virus-induced metabolic changes in the liver, including amino-acid-related pathways, correlated with the infection status of the mice and were independent of altered food intake (Figures 1H and 2D).

IFN-I Signaling Reprograms Hepatic Metabolism

The observed changes of hepatic amino acid metabolism became noticeable as early as 2 days, which coincides with peak serum levels of IFN-α and IFN-β in mice infected with LCMV (Bhattacharya et al., 2015). Thus, we aimed to dissect a potential role of early IFN-I signaling in the observed changes in the liver. To measure the impact of IFN-I signaling on liver parenchyma, we performed an extracellular metabolic flux analysis of primary murine hepatocytes upon stimulation with IFN-β *in vitro*. IFN-β affected proxies for mitochondrial respiration and glycolysis in an IFNAR1-dependent manner (Figures 3A and 3B), indicating that IFN-I signaling affects metabolism of hepatocytes. To deconvolute tissue heterogeneity *in vivo* and to assess the contribution of IFN-I signaling to metabolic reprogramming in the liver, we took advantage of a genetic model of hepatocyte-specific ablation of *Ifnar1* (*Alb-Cre ERT2 Ifnar1*$^{fl/fl}$ [*Ifnar1*$^{\Delta/\Delta}$]) and respective littermate controls (*Alb-Cre ERT2 Ifnar1*$^{+/+}$ [*Ifnar1*$^{+/+}$]). These mice did not reveal any genotype-specific differences in viremia or serum concentration of IFN-α 1.5 days after infection (Figures S3A–S3C), suggesting that any potential differences seen in expression profiling of liver tissue from *Ifnar1*$^{\Delta/\Delta}$ versus *Ifnar1*$^{+/+}$ mice is unlikely to be due to altered viral loads and/or systemic IFN-I responses. Moreover, naive and infected *Ifnar1*$^{\Delta/\Delta}$ versus *Ifnar1*$^{+/+}$ animals displayed comparable abundances of immune-cell-related transcripts, suggesting only minor differences of immune cell infiltration between the genotypes at this early time point after infection (Figure S3D). Next, we analyzed transcriptomic changes of liver tissue taken from uninfected and infected mice of either genotype at the peak of serum IFN-α levels and employed a limma (2×2 factorial) interaction model. This resulted in a set of 526 hepatocyte-intrinsic IFNAR1-regulated genes, which were found to be associated with both classical ISG responses as well as metabolic processes (Figures 3C, 3D, and S3E; Table S5A). The regulated genes could be divided into two major classes—IFNAR1-stimulated (cluster 1) and IFNAR1-repressed (cluster 2) genes (Figure 3C; Table S5B). The majority of induced genes were well-known classical ISGs encoding for antiviral effectors (cluster 1; Figure 3D; Table S5C; Schoggins et al., 2011). Interferon-repressed genes (IRGs), which are not that well characterized (Mostafavi et al., 2016; Schoggins et al., 2011), were found to be strongly enriched for metabolism-associated processes (cluster 2; Figure 3D; Table S5C). In addition, clusters 3 and 4 contained genes whose maintained expression depended on intact IFNAR1 signaling and were also associated with metabolic processes (Figure 3D; Table S5C). Hepatocyte-intrinsic IFNAR1 signaling mainly regulated metabolic genes on day 2 after infection. Notably, a bioinformatic intersection with our longitudinal data obtained from chronically infected wild-type mice suggested that the IFN-I-dependent regulation of many of these genes is maintained beyond these early time points (Figure 3E). Specifically, virus-induced gene regulation of amino-acid-related pathways (Figure 1) was driven by hepatocyte-intrinsic IFNAR1 signaling (Figure 3E) and corresponded with the differentially regulated metabolites observed in infected wild-type mice (Figure 1H). To investigate whether these metabolic changes depended on hepatocyte-intrinsic IFNAR1 signaling, we performed metabolomics and found that systemic serum levels were indeed regulated by local IFNAR1 signaling of hepatocytes (Figure S3F). In a more stringent analysis using the limma (2×2 factorial) interaction model, we found 15 serum metabolites to be regulated by hepatocyte-intrinsic IFNAR1 signaling, including the semi-essential amino acid arginine and its downstream metabolite ornithine (Figure 3F). In summary, our data indicate that hepatocyte-intrinsic IFNAR1 signaling acts as a transcriptional regulator of liver metabolism and results in changes of circulating metabolites during infection.

Hepatocyte-Intrinsic *Ifnar1* Signaling Breaks the Urea Cycle

Arginine and ornithine are both key metabolites of the urea cycle. The involved enzymes are encoded by *Arg1*, *Otc*, and

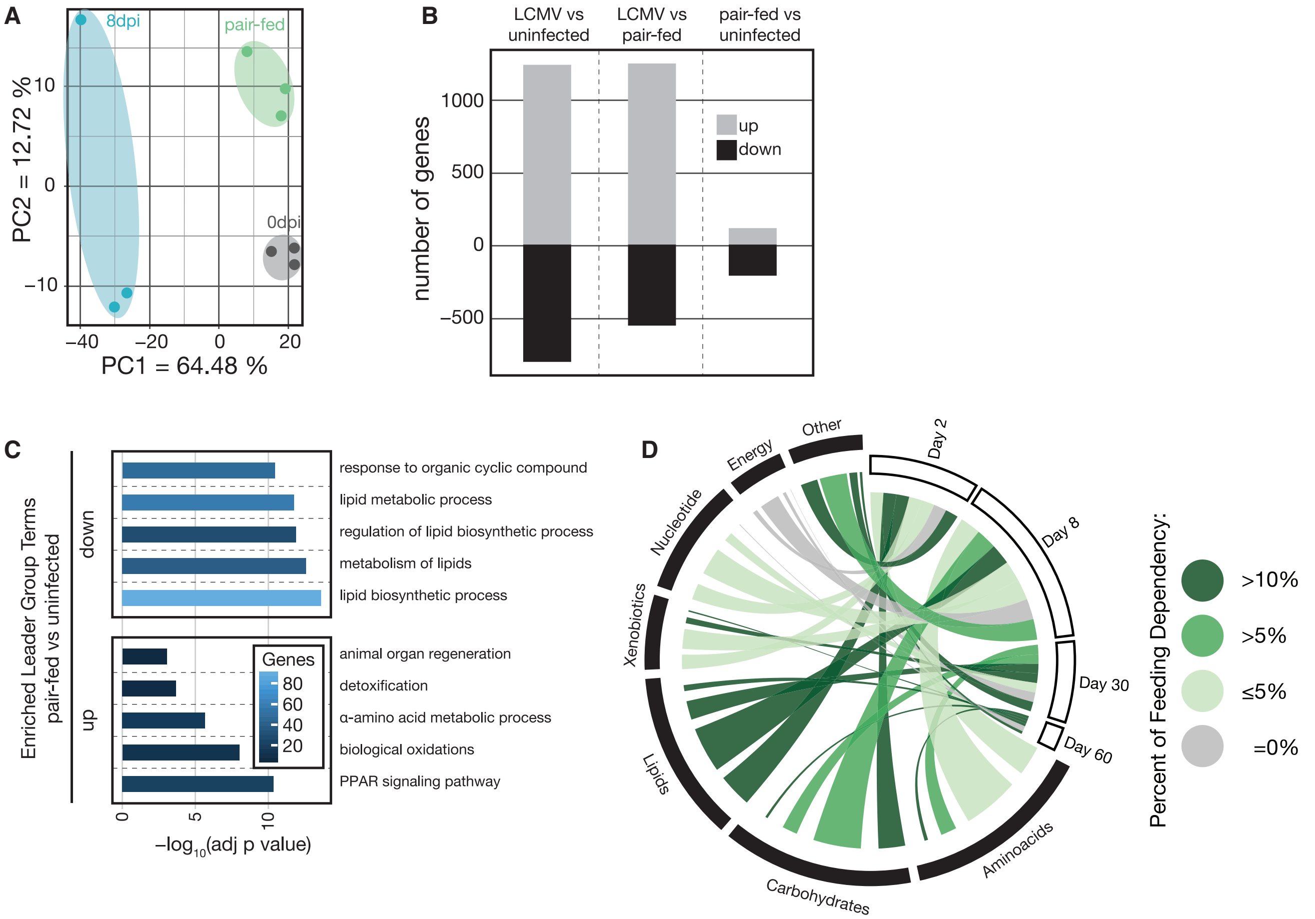

Figure 2. Reduced Food Intake upon LCMV Infection Mainly Affects Lipid-Metabolism-Associated Genes in the Liver

(A) PCA of liver transcriptomes of naive, 8 days after LCMV infection, and pair-fed (8 days) mice (n = 3).

(B) Significantly deregulated transcripts in liver tissue upon LCMV infection at 8 days after infection compared to naive and pair-fed and between pair-fed and naive animals (n = 3).

(C) Enriched GO terms and pathways (ClueGO) of significantly up- and downregulated genes of pair-fed versus naive mice (n = 3).

(D) Significantly regulated metabolic genes in the livers of pair-fed versus naive animals super-imposed on the previously identified significantly regulated metabolic transcripts and proteins at the indicated time points after LCMV infection (based on Figure 1G).

For (A)–(C), transcriptomic data are derived from one experiment. See also Figure S2.

Ass1, which are primarily expressed in the liver, as well as *Asl*, which is ubiquitously expressed (Uhlén et al., 2015). Expression analysis of liver tissue of uninfected and infected *Ifnar1*$^{\Delta/\Delta}$ and *Ifnar1*$^{+/+}$ mice highlighted that expression of *Otc* and *Ass1* was repressed in a hepatocyte-intrinsic IFNAR1-dependent manner (Figures 4A, S4A, and S4B). *Arg1* was induced, whereas *Asl* was repressed upon infection, both independent of hepatocyte-intrinsic IFNAR1 signaling (Figure 4A). These infection-induced transcriptional changes of the urea cycle genes *Otc*, *Ass1*, *Asl*, and *Arg1* were maintained on transcriptomic and proteomic level up to 30 days after infection, with the most pronounced effects seen on days 2 and 8 (Figures S4C–S4F). Of note, the regulation of *Otc* and *Ass1* was independent of food intake (Figure S4G).

To corroborate the hepatocyte-specific origin of the observed reprogramming of the hepatic urea cycle, we performed single-cell RNA-seq (scRNA-seq) of primary murine hepatocytes isolated from naive or LCMV-infected wild-type mice 2 days after infection. Both samples showed comparable numbers of reads (uninfected: 1,493.5; infected: 1,429.0) and genes (uninfected: 705.5; infected: 761.0) per cell. We identified 348 differentially expressed genes in hepatocytes that correlated well with changes observed in bulk liver tissue (Figures 1, S4H, and S4I). Accordingly, genes induced in hepatocytes were associated with antiviral innate immune responses, whereas repressed genes were vastly associated with metabolic processes (Figures S4J and S4K). Hepatocytes clustered by the infection status of the mice (Figure 4B), and we confirmed hepatocyte-intrinsic repression of *Ass1* and *Otc* upon infection, which inversely correlated with expression levels of the ISG *Ifit1* on the single-cell level (Figures 4C and 4D). Transcriptional repression of *Otc* and *Ass1* manifested in reduced abundance of OTC and ASS1 proteins in hepatocytes as observed by immunohistochemistry on days 2 and 8 after infection (Figures 4E and 4F). As expected, STAT1, a master regulator of IFNAR1 downstream signaling, was significantly upregulated in hepatocytes (Figures 4E and 4F). The modulation of

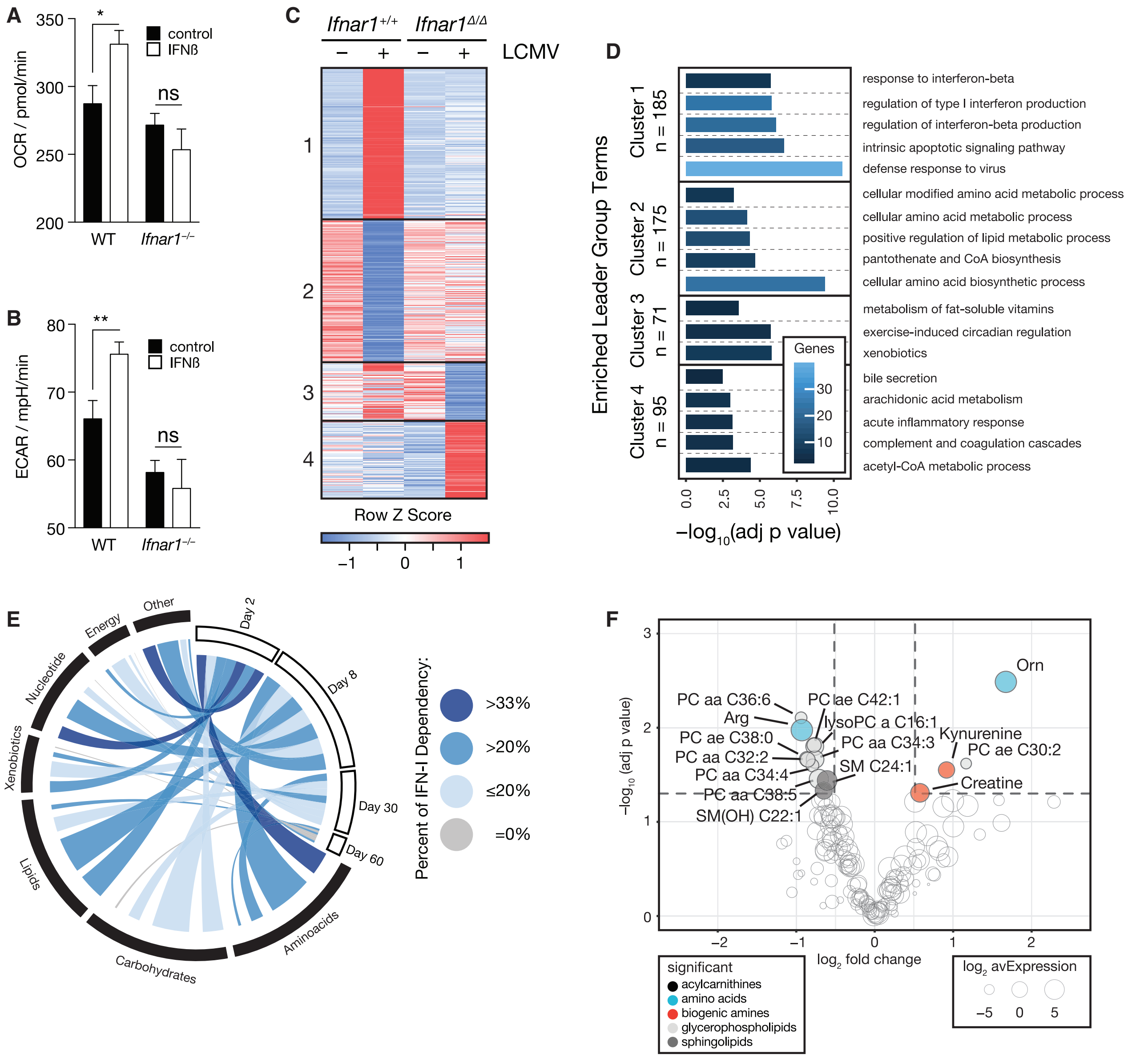

Figure 3. Hepatocyte-Intrinsic IFNAR1 Signaling Is a Transcriptional Regulator of Liver Metabolism and Shapes Systemic Metabolism

(A and B) Oxygen consumption rate (OCR) (A) and extracellular acidification rate (ECAR) (B) of wild-type and *Ifnar1*$^{-/-}$ primary hepatocytes treated for 4 h with IFN-β (n = 11).

(C) Clustering by expression profile (FPKM; k-means; Pearson's correlation) of transcripts significantly regulated (limma interaction model) by hepatocyte-intrinsic IFNAR1 signaling (n = 3).

(D) Enriched GO terms and pathways (ClueGO) of transcripts identified in the groups clusters in (C).

(E) Metabolism-associated transcripts significantly regulated by hepatocyte-intrinsic IFNAR1 signaling super-imposed on the previously identified significantly regulated metabolic transcripts and proteins at the indicated time points after LCMV infection (based on Figure 1G).

(F) Serum metabolites significantly regulated (limma interaction model) by hepatocyte-intrinsic IFNAR1 signaling in naive and LCMV-infected animals (n = 3).

For (A) and (B), data for one out of two representative experiments are shown. For (C), (D), and (F), transcriptomic and metabolomic data are derived from one experiment. Symbols represent the arithmetic mean ± SEM; *p < 0.05; **p < 0.01 (Student's t test). See also Figure S3.

Otc and *Ass1* on day 2 and day 8 after infection was confirmed by real-time PCR in sorted hepatocytes (Figure 4G).

In line with this, liver tissue of infected mice exhibited reduced enzymatic activity of OTC and ASS1, which we measured by the abundance of the ^{13}C-labeled reaction products argininosuccinate (ASS1) or citrulline (OTC) after pulsing with ^{13}C-labeled substrates. Addition of the ASS1-specific inhibitor α-methyl-DL-aspartic acid (MDLA) abolished production of argininosuccinate and indicated specificity of the assay (Figure S4L). In line with the IFNAR1-dependent increase of systemic ornithine levels upon infection (Figure 4A), these data indicate that IFNAR1 represses the degradation of ornithine via OTC in hepatocytes.

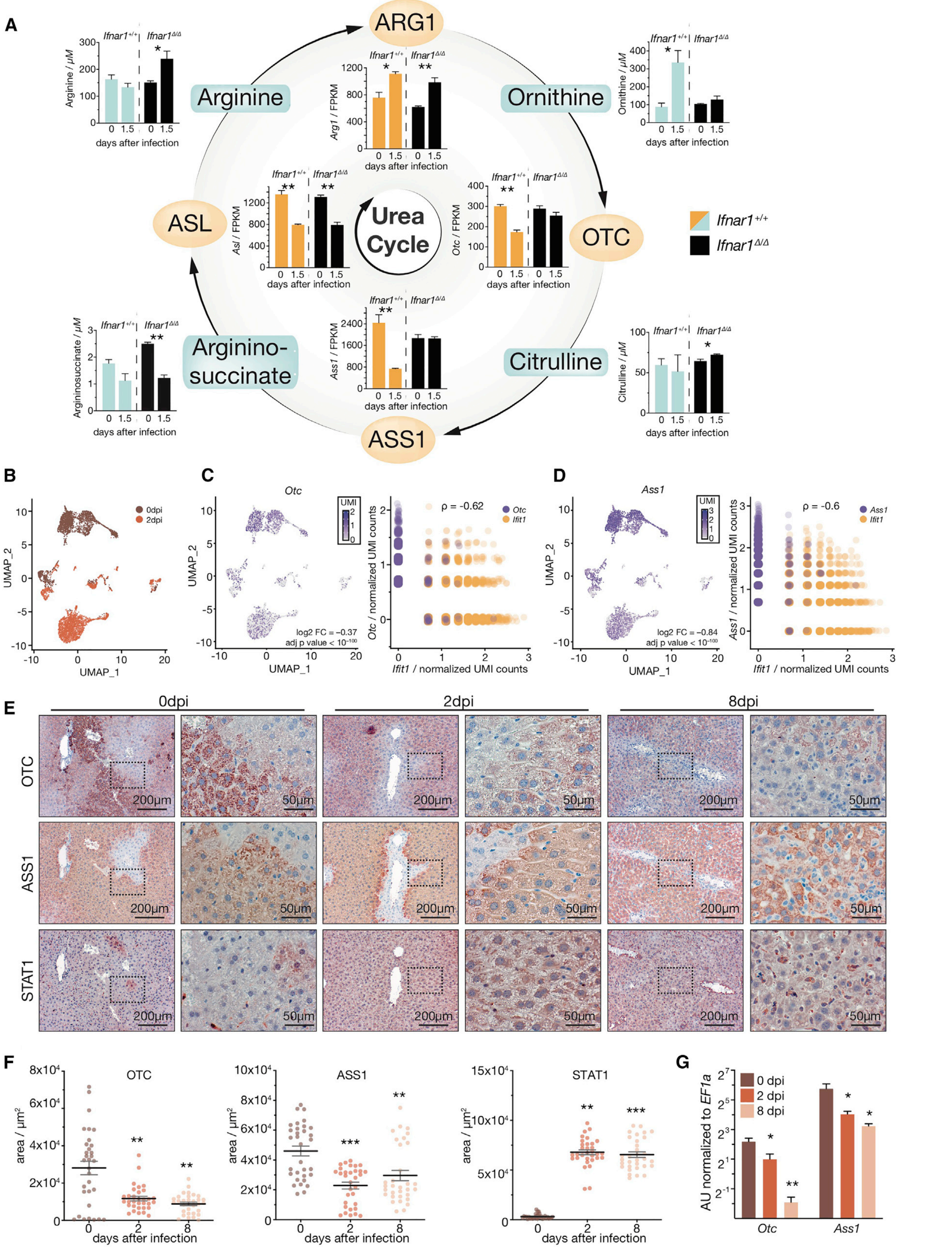

(legend on next page)

The downregulation of *Asl* expression, together with decreased argininosuccinate, is expected to aggravate the infection-induced break in the urea cycle by limiting arginine re-synthesis (Figure 4A).

We confirmed the aforementioned transcriptional changes of the urea cycle and upregulation of *Ifit1* in the viral infection model of coronavirus mouse hepatitis virus (MHV) by real-time PCR (Figure S4M). To test whether similar responses in the liver are seen in an inflammatory context, we administered the synthetic viral RNA analog and TLR3 agonist polyinosinic-polycytidylic acid (poly(I:C)) intraperitoneally to wild-type mice. Poly(I:C) treatment resulted in strong induction of *Ifit1* and corroborated the upregulation of *Arg1* and downregulation of *Otc* and *Ass1* in liver tissue by real-time PCR (Figure S4N). Treatment with 100 ng recombinant IFN-α induced an ISG signature in the liver but did not yield reproducible changes of expression of these genes (data not shown), suggesting that interferon signaling is required, but not sufficient to regulate the urea cycle.

Next, we aimed to address whether viral infection leads to hyperammonemia, a hallmark parameter of urea cycle deficiency (UCD). Indeed, blood ammonia levels were significantly increased from 2 up to 8 days after LCMV infection, correlating with downregulation of the ammonia-fixating enzyme *Cps1* (Figures 5A and S5A). This prompted us to further characterize the observed urea-cycle-related metabolic changes on a systemic level by *in vivo* tracing of $^{13}C_6$ heavy-isotope-labeled arginine (Figure 5B). Liver tissue of infected mice that received a bolus of $^{13}C_6$ arginine showed increased concentration of labeled ornithine and citrulline (Figure 5C). Arginine was not detected in the liver as reported previously (Wu et al., 2009). These local metabolic changes in the liver tissue, together with transcriptomic and proteomic changes in hepatocytes highly correlated with changes in systemic metabolism. We detected increased $^{13}C_6$ arginine degradation in the serum (Figure 5D), which is consistent with the induction of *Arg1* (Figures 4A and S4C). Together with IFNAR1-dependent repression of *Otc* and *Ass1* (Figures 4A–4D), this resulted in the systemic accumulation of $^{13}C_5$ ornithine and $^{13}C_5$ citrulline, respectively (Figure 5D). In accordance with increased arginine degradation and decreased ornithine degradation, we detected elevated levels of $^{13}C_1$-labeled urea in liver tissue (Figure S5B), whereas $^{13}C_1$-labeled urea was only mildly elevated in the circulation (Figure S5C). Together with the reduction of $^{13}C_5$ arginine, the end product of this pathway, these results support the notion that viral infection initiates a break of the hepatic urea cycle as early as 2 days after infection that persists at least until 8 days after infection and alters levels of metabolites in the circulation (Figure S5D).

Consistently, we observed a significant decrease of the arginine-to-ornithine ratio in the serum of mice infected with LCMV as a result of both reduced arginine and increased ornithine levels (Figures 5E and S5E). Similar changes in serum concentration of arginine and ornithine were seen upon infection with MHV (Figures S5F and S5G). Taken altogether, our results derived from different experimental models demonstrate conserved transcriptional and metabolic modulation of the urea cycle upon viral infection.

Systemic Arginine-Ornithine Homeostasis Is a Regulator of Antiviral Adaptive Immunity

Because activated T cells are auxotrophic for arginine (Geiger et al., 2016; Murray, 2016), we hypothesized that an altered arginine-to-ornithine serum ratio might exert immunomodulatory function. To test this *in vitro*, primary naive murine splenic CD8 T cells were activated with anti-CD3/CD28 antibodies for 3 days and IFN-γ and tumor necrosis factor alpha (TNF-α) production was assessed by flow cytometry. CD8 T cells cultured in medium with 11.5 μM arginine, a concentration comparable to the observed changes *in vivo*, displayed reduced cytokine production compared to standard cell culture medium containing 1,150 μM arginine (Figure 6A). Addition of ornithine, which is absent in standard cell culture medium, resulted in an additive suppressive effect on cytokine production (Figure 6A). This indicated that decreased concentrations of arginine impact CD8 T cell responses and that these effects are aggravated by a simultaneous increase of ornithine.

To experimentally uncouple the observed changes in the urea cycle from other co-occurring effects *in vivo*, we treated wild-type mice with recombinant pegylated human arginase 1 (recArg1) (Cheng et al., 2007) to aggravate the observed endogenous regulation of arginine and ornithine in the circulation. As expected, recArg1 converted arginine to ornithine in the serum and closely recapitulated the decreased arginine-to-ornithine ratio seen upon viral infection (Figures 5E and S6A). Administration of recArg1 did not affect the abundance of splenic T cells of infected and uninfected animals on day 8 after infection (Figure S6B). Yet, we recognized an impaired shift from naive (CD62L$^+$CD44$^-$) to effector (CD62L$^-$CD44$^+$) T cells (Figures S6C and S6D). Further, the numbers of virus-specific CD8

Figure 4. IFNAR1 Contributes to Infection-Induced Metabolic Reprogramming of Hepatocytes

(A) Depiction of the urea cycle, expression, and concentrations of the associated genes and serum metabolites in naive and LCMV-clone-13-infected *Alb-Cre ERT2 Ifnar1*$^{fl/fl}$ (*Ifnar1*$^{\Delta/\Delta}$) and *Ifnar1*$^{+/+}$ mice (n = 3).

(B) Uniform Manifold Approximation and Projection (UMAP) clustering of single-cell RNA-seq data of primary hepatocytes sorted from naive or infected wild-type animals 2 days after LCMV infection.

(C and D) Expression levels and correlation with *Ifit1* levels (IFN-I-stimulated gene) of (C) *Otc* and (D) *Ass1* in hepatocytes isolated from naive versus infected animals (n = 2; pooled for each condition). Each dot represents a single hepatocyte.

(E) Representative immunohistochemical staining of OTC, ASS1, and STAT1 in liver sections of naive or LCMV-infected (2 and 8 days after infection) wild-type mice.

(F) Quantification of immunohistochemical staining of OTC, ASS1, and STAT1 using HistoQuest software (n = 4 and 8 pictures per mouse were quantified).

(G) Expression of *Otc* and *Ass1* in sorted primary hepatocytes isolated from naive and LCMV-infected (2 and 8 days after infection) mice (n = 3).

For metabolite data in (A), one of two representative experiments is shown. For (B)–(G), single-cell transcriptomic and histological data are derived from one experiment. Symbols represent the arithmetic mean ± SEM; *p < 0.05; **p < 0.01; ***p < 0.001 (Student's t test). Log 2 fold changes and adjusted p values for single-cell RNA-seq data were computed as described in the STAR Methods. See also Figure S4.

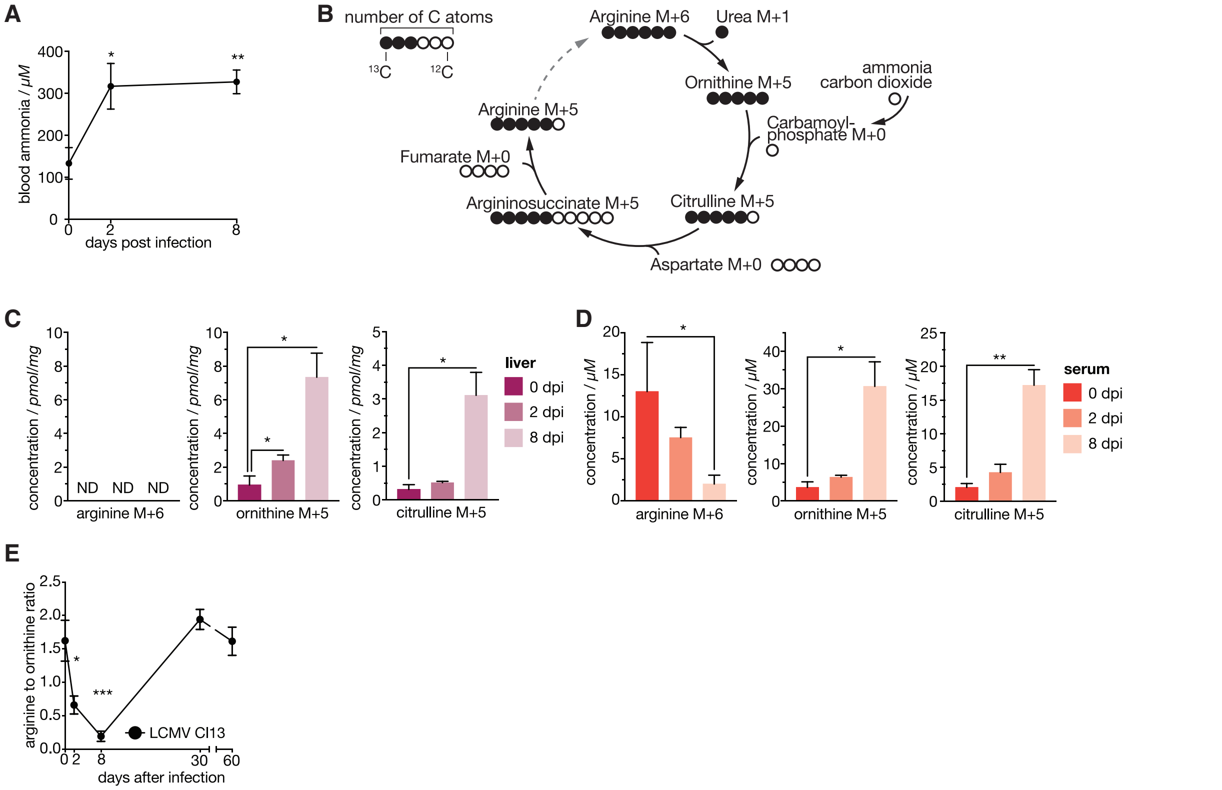

Figure 5. Viral Infection Reprograms the Hepatic Urea Cycle to Shape Systemic Metabolism

(A) Blood ammonia upon LCMV-clone-13-infected wild-type mice (n = 5–6).

(B) Schematic depiction of the carbon flow in urea cycle starting from $^{13}C_6$ arginine illustrating ^{13}C-labeled (full circles) and unlabeled ^{12}C atoms of the respective metabolites.

(C) Concentrations of $^{13}C_6$-labeled arginine, $^{13}C_5$-labeled ornithine, and $^{13}C_5$-labeled citrulline in liver tissue of naive and LCMV-infected (2 and 8 days after infection) mice (n = 3–6).

(D) Concentrations of $^{13}C_6$-labeled arginine, $^{13}C_5$-labeled ornithine, and $^{13}C_5$-labeled citrulline in serum of naive and LCMV-infected (2 and 8 days after infection) mice (n = 3–6).

(E) Systemic arginine-to-ornithine ratio of LCMV-infected (n = 8) wild-type animals.

For (A), one of two representative experiments is shown. For (E), data are pooled from two independent experiments. For (C) and (D), metabolite tracing data are derived from one experiment. ND, not detected. Symbols represent the arithmetic mean ± SEM; *$p < 0.05$; **$p < 0.01$; ***$p < 0.001$ (Student's t test). See also Figure S5.

T cells (Figure 6B) and their ability to produce the cytokines IFN-γ and TNF-α upon peptide re-stimulation (Figures 6C–6E) were diminished upon recArg1 treatment. Virus-specific CD4 T cells were similarly affected (Figure S6E). CD8 T cell effector function remained impaired in recArg1-treated animals at least up to 50 days after infection (Figure 6F) and coincided with elevated viral loads in blood (Figure 6G) and organs 8 and 50 days after infection (Figures S6F–S6I). To investigate whether impaired cytokine production of CD8 T cells upon recArg1 treatment coincides with exhaustion, we analyzed PD1 expression on virus-specific CD8 T cells in the blood of mice 30 days after infection. This did not reveal any obvious differences for PD1 upon treatment with recArg1 (Figure S6J). Strikingly, treatment with recArg1 significantly ameliorated virus-induced CD8 T-cell-mediated tissue damage as determined by reduced serum concentration of ALT and AST (Figures 6H and 6I). We conclude that modulation of the systemic arginine-to-ornithine ratio regulates antiviral T cell responses to ameliorate virus-induced tissue damage.

DISCUSSION

Metabolic adjustments are crucial for activation and differentiation of immune cells (Jha et al., 2015; Pearce and Pearce, 2013; Pearce et al., 2009). Our study identified an IFNAR1-dependent mechanism whereby hepatocytes receive instructions to repress the transcription of genes with metabolic function. The role of IFN-repressed genes in disease and homeostasis is still poorly understood. Yet it is noticeable that IFN-I-driven expression signatures from other organs and cell types do not appear to feature similar global repressive gene regulation (Mostafavi et al., 2016; Schoggins et al., 2011). This potentially highlights a unique feature of the liver, which rapidly integrates inflammatory cues from the environment to modulate its intrinsic metabolism and, as a consequence, its systemic metabolic output to peripheral organs. This IFNAR1-mediated metabolic reprogramming of the liver may give rise to an unfavorable environment for pathogens (Sanchez et al., 2018), optimize the metabolic milieu for mounting adaptive immune responses (Ma et al., 2017), or

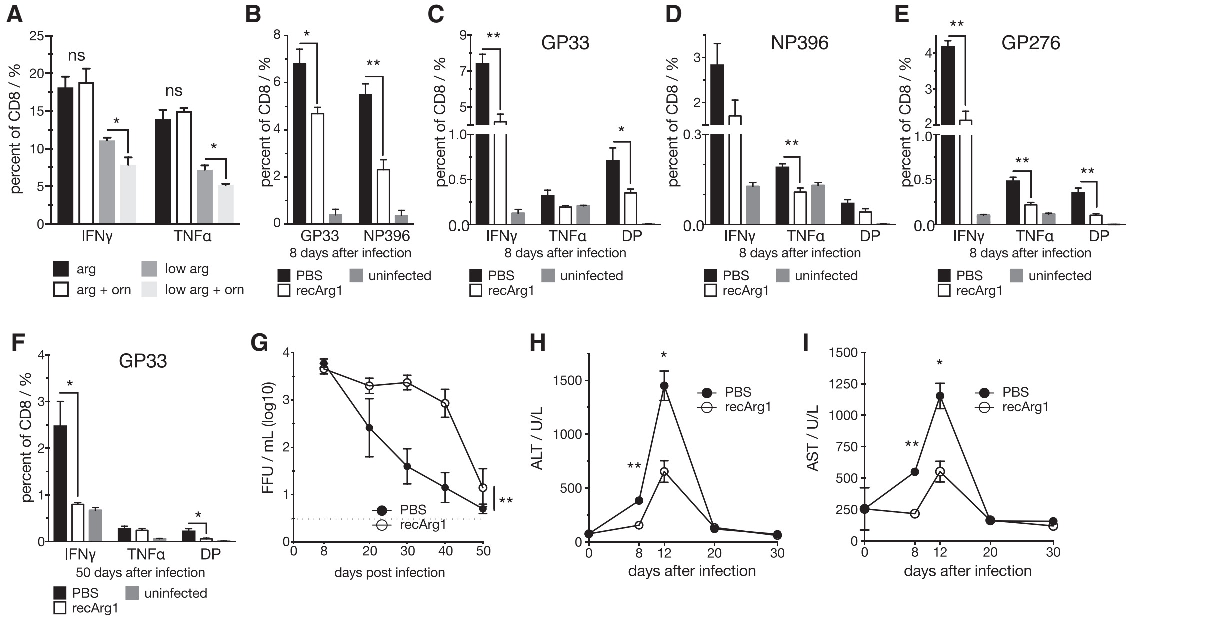

Figure 6. Arginase 1 Treatment Reduces Antiviral CD8 T Cell Responses and Ameliorates Virus-Induced Hepatitis during LCMV Cl13 Infection

(A) IFN-γ and TNF-α production of primary murine splenic CD8 T cells cultured in medium containing 1,150 μM (standard RPMI 1640 concentration) or 11.5 μM L-arginine with or without 1,150 μM L-ornithine upon CD3/28 activation for 3 days (n = 4).

(B) GP33- and NP396-specific splenic tetramer$^+$ CD8 T cells (n = 5).

(C–E) (C) GP33, (D) NP396, (E) GP276 virus-specific IFN-γ and TNF-α producing virus-specific splenic CD8 T cells upon recArg1 treatment 8 days after LCMV clone 13 infection (n = 5).

(F) IFN-γ and TNF-α producing splenic GP33-specific CD8 T cells in mice treated with recArg1 twice per week up to 50 days after infection (n = 5).

(G) Viral load in blood upon recArg1 treatment (n = 5).

(H and I) Serum ALT (H) and AST (I) levels upon recArg1 treatment (n = 5).

Data in (A)–(I) are representative for one of at least two experiments. Symbols represent the arithmetic mean ± SEM. Dotted line implicates limit of detection. *$p < 0.05$; **$p < 0.01$ (Student's t test, A–F, H, and I, or two-way ANOVA, G). See also Figure S6.

instruct tissue repair and regeneration to prevent extensive tissue pathology. None of these options are mutually exclusive, and host damage control may be achieved by modulating disease tolerance and/or reducing immune effectors (Medzhitov, 2008).

Next to other metabolic changes, we identified a regulatory effect of IFNAR1 signaling on the urea cycle, a central metabolic pathway that is primarily expressed in the liver. Its primary function is the conversion of circulating toxic ammonia into non-toxic urea with a fumarate shunt connecting the urea cycle to the TCA cycle (Meijer et al., 1990). Fumarate can subsequently be metabolized to aspartate and α-ketoglutarate, two major precursors of *de novo* amino acid synthesis. Hence, the urea cycle is a central metabolic pathway that is crucial for both detoxication as well as cellular proliferation (Watford, 2003).

We modeled the altered levels of serum arginine and ornithine upon viral infection by administration of recombinant arginase and demonstrate a suppressive effect on virus-specific CD8 T cell responses. This is in line with nutrient availability as an important factor that may either boost or dampen effector functions of T cells (Chang et al., 2015; Geiger et al., 2016; Johnson et al., 2018; Ma et al., 2017; Sinclair et al., 2013; Van de Velde and Murray, 2016). A recent study identified that T cells may actively shape serum metabolite levels themselves, thereby creating potential functional feedback loops between systemic metabolism and T cell activation (Miyajima et al., 2017). This study adds another regulatory layer next to well-established mechanisms, including T cell exhaustion, of how the host may fine-tune adaptive immunity during chronic infection (Wherry and Kurachi, 2015).

The urea cycle is linked to amino acid, polyamine, and nucleotide metabolic pathways and represents a major hub of systemic nitrogen metabolism, which contributes to the formation of advanced liver disease (Li et al., 2019). Accordingly, inherited urea cycle disorders (UCDs) and the resulting hyperammonemia with altered serum levels of the associated metabolites display severe health threats (Mew et al., 2003). We found that mice infected with LCMV display hyperammonemia, a hallmark of hepatic encephalopathy (Tranah et al., 2013). Additional investigations will be required to address whether infection-associated hyperammonemia may modulate sickness behavior and influence disease progression (Dantzer et al., 2008; Wang et al., 2019).

Downregulation or even loss of *Otc* and *Ass1* are common features of many cancers, limiting aspartate consumption by the urea cycle and instead feeding into *de novo* nucleotide synthesis associated with tumor growth (Feun et al., 2012; Lam et al., 2011; Lee et al., 2018; Rabinovich et al., 2015). *Otc* and *Ass1* are also crucial for *de novo* arginine synthesis. As a result, many tumors are auxotrophic for arginine, which renders

arginine a metabolic vulnerability that is currently therapeutically exploited by recArg1 treatment of cancer patients (De Santo et al., 2018). In line, UCDs and in particular *Ass1* deficiency of tumors potentially synergize with checkpoint blockade treatment in tumors (Lee et al., 2018). Interestingly, reduced serum levels of arginine are associated with less liver tissue damage in chronic HBV infection although increased serum levels of arginine and arginine derivatives have been associated with non-alcoholic fatty liver disease (Dumas et al., 2014; Li et al., 2011; Pallett et al., 2015; Soga et al., 2011). Finally, deregulated ornithine metabolism and increased synthesis of polyamines may also impact cellular proliferation as well as the replication of diverse groups of viruses, potentially impacting on infection-associated cancer formation and progression (Casero et al., 2018; Mounce et al., 2016).

Using different approaches, we demonstrate that virus-induced IFNAR1 signaling in hepatocytes leads to downregulation of *Otc* and *Ass1*. The simultaneous upregulation of *Arg1* during viral infection of the liver suggests that, during infection, hepatocytes are reprogrammed toward increased arginine degradation and ornithine accumulation. However, we cannot exclude the possibility that infiltrating immune cells, including myeloid-derived suppressor cells (MDSCs), may contribute to increased *Arg1* expression in the liver (Gabrilovich, 2017). In line with this, our $^{13}C_6$ heavy-isotope-labeling experiments confirmed that viral infection increases the hepatic conversion of arginine to ornithine and correlates with systemic accumulation of ornithine and citrulline. The observed infection-dependent reduction of the urea cycle end product $^{13}C_5$ arginine further supported the notion of an IFNAR1-mediated break of the urea cycle. Still, these results do not rule out the potential additional contributions of extrahepatic events, as changes in the endocrine system are likely co-occurring with systemic metabolic reprogramming and/or sickness behavior during infection. Previous studies also established a link between hepatic autophagy, hepatocyte-derived circulating ARG1, and the systemic arginine-to-ornithine ratio, leading to reduced tumor growth in mice (Poillet-Perez et al., 2018). These lines of evidence suggest that endogenous metabolic reprogramming of the urea cycle modulates the pathophysiology during infectious disease. Our results may also provide a potential explanation for why many non-hepatotropic infections result in concomitant hepatitis (Adams and Hubscher, 2006; Lalazar and Ilan, 2014). Taken together, our study provides evidence for an infection-induced reprogramming of the urea cycle in hepatocytes, a central metabolic pathway in the liver, leading to hyperammonemia alongside with the deregulation of systemic metabolism. We identified hepatocyte-intrinsic IFNAR1 signaling as a key regulator of circulating levels of arginine and ornithine to fine-tune adaptive immunity. This endogenous cytokine-driven mechanism of immunomodulation and host protection across organs could be targeted to ameliorate tissue pathology in infectious and inflammatory diseases.

STAR★METHODS

Detailed methods are provided in the online version of this paper and include the following:

- KEY RESOURCES TABLE
- LEAD CONTACT AND MATERIALS AVAILABILITY
- EXPERIMENTAL MODEL AND SUBJECT DETAILS
  - Mice
  - Viruses and cell lines
- METHOD DETAILS
  - Infections
  - Pharmacological perturbations
  - Conditional Ifnar1 ablation on hepatocytes
  - poly(I:C) treatment
  - Isolation of primary murine hepatocytes
  - Metabolite tracing
  - Enzymatic assays
  - Gene expression analyses of sorted primary murine hepatocytes
  - Immunohistochemistry
  - Cytokine determination
  - Blood chemistry
  - RNA isolation and real-time PCR
  - Metabolic flux measurements
  - CD8 T cell isolation and *in vitro* activation
  - Flow cytometry
  - Quantitative proteomics
  - Targeted LC-MS based metabolite measurements
- QUANTIFICATION AND STATISTICAL ANALYSIS
  - RNA-seq data processing
  - Single cell RNA-seq processing
  - Principal component analysis
  - Hierarchical clustering
  - Interaction model
  - Enrichments and pathway analyses
  - Mass spectrometry data processing
  - Targeted LC-MS based metabolite quantification
  - Metabolite tracing quantification
  - Immunohistochemistry quantification
  - Statistical information
- DATA AND CODE AVAILABILITY

SUPPLEMENTAL INFORMATION

Supplemental Information can be found online at https://doi.org/10.1016/j.immuni.2019.10.014.

A video abstract is available at https://doi.org/10.1016/j.immuni.2019.10.014#mmc8.

ACKNOWLEDGMENTS

We want to thank Sarah Niggemeyer, Jenny Riede, Sabine Jungwirth, Nicole Fleischmann, and Patricia Schittenhelm for animal caretaking; Safia Zahma for histopathology and immunohistochemistry processing; Thomas Penz, Michael Schuster, Martin Senekowitsch, Daniele Barreca, and Christoph Bock from the Biomedical Sequencing Facility at CeMM; Katja Parapatics from the Proteomics Facility at CeMM; Jacques Colinge for initial data analyses; and Christoph J. Binder from CeMM and Medical University of Vienna and Roger Geiger from the Institute for Research in Biomedicine, Bellinzona, for support and feedback. The following reagents were obtained through the NIH Tetramer Core Facility: LCMV MHCI tetramers GP33 (in Phycoerythrin [PE]) and NP396 (in Allophycocyanin [APC]). This project was funded with support of the European Research Council (ERC) under the European Union's Horizon 2020 research and innovation program (grant agreement no. 677006, "CMIL" to A. Bergthaler). A.L., A. Bhattacharya, and J.S.B. were supported by a DOC fellowship of the Austrian Academy of Sciences. N.P. was supported

by an Ambizione grant awarded by the Swiss National Science Foundation (PZ00P3_180011/1).

AUTHOR CONTRIBUTIONS

A.L., A.M.P., and A. Bergthaler wrote the manuscript. A.L. designed and performed *in vivo* and *in vitro* studies. A. Bhattacharya, M.F.S., B.A., L.K., T.P., J.-W.G., and N.P. performed *in vitro* and/or *in vivo* experiments. D.V., P.M., and K.L.B. contributed to proteomic analyses. A.L., A.M.P., M.C., and J.M. performed bioinformatic analyses. K.K., B.G., D.R., and M.O.-K. performed metabolite measurements. A.O. performed histological analyses. U.K. and P.N.C. provided reagents. H.B., G.S., J.S.B., B.L., R.M., and M.T. analyzed data and provided feedback. A. Bergthaler supervised the study and designed and analyzed experiments.

DECLARATION OF INTERESTS

P.N.C. is the founder of Biocancer Treatment International, and G.S. is scientific advisor of Biocancer Treatment International.

Received: May 22, 2019
Revised: September 10, 2019
Accepted: October 25, 2019
Published: November 26, 2019

REFERENCES

Adams, D.H., and Hubscher, S.G. (2006). Systemic viral infections and collateral damage in the liver. Am. J. Pathol. *168*, 1057–1059.

Mew, N.A., Simpson, K.L., Gropman, A.L., Lanpher, B.C., Chapman, K.A., and Summar, M.L. (2003). Urea cycle disorders overview. In GeneReviews, M.P. Adam, H.H. Ardinger, and R.A. Pagon, et al., eds. (University of Washington).

Baazim, H., Schweiger, M., Moschinger, M., Xu, H., Scherer, T., Popa, A., Gallage, S., Ali, A., Khamina, K., Kosack, L., et al. (2019). $CD8^+$ T cells induce cachexia during chronic viral infection. Nat. Immunol. *20*, 701–710.

Bennett, K.L., Funk, M., Tschernutter, M., Breitwieser, F.P., Planyavsky, M., Ubaida Mohien, C., Muller, A., Trajanoski, Z., Colinge, J., Superti-Furga, G., and Schmidt-Erfurth, U. (2011). Proteomic analysis of human cataract aqueous humour: Comparison of one-dimensional gel LCMS with two-dimensional LCMS of unlabelled and iTRAQ®-labelled specimens. J Proteomics *74*, 151–166.

Bhattacharya, A., Hegazy, A.N., Deigendesch, N., Kosack, L., Cupovic, J., Kandasamy, R.K., Hildebrandt, A., Merkler, D., Kühl, A.A., Vilagos, B., et al. (2015). Superoxide dismutase 1 protects hepatocytes from type I interferon-driven oxidative damage. Immunity *43*, 974–986.

Bindea, G., Mlecnik, B., Hackl, H., Charoentong, P., Tosolini, M., Kirilovsky, A., Fridman, W.-H., Pagès, F., Trajanoski, Z., and Galon, J. (2009). ClueGO: a Cytoscape plug-in to decipher functionally grouped gene ontology and pathway annotation networks. Bioinformatics *25*, 1091–1093.

Breitwieser, F.P., and Colinge, J. (2013). Isobar(PTM): a software tool for the quantitative analysis of post-translationally modified proteins. J Proteomics *90*, 77–84.

Breitwieser, F.P., Müller, A., Dayon, L., Köcher, T., Hainard, A., Pichler, P., Schmidt-Erfurth, U., Superti-Furga, G., Sanchez, J.-C., Mechtler, K., et al. (2011). General statistical modeling of data from protein relative expression isobaric tags. J. Proteome Res. *10*, 2758–2766.

Buck, M.D., Sowell, R.T., Kaech, S.M., and Pearce, E.L. (2017). Metabolic instruction of immunity. Cell *169*, 570–586.

Casero, R.A., Jr., Murray Stewart, T., and Pegg, A.E. (2018). Polyamine metabolism and cancer: treatments, challenges and opportunities. Nat. Rev. Cancer *18*, 681–695.

Cervantes-Barragan, L., Züst, R., Weber, F., Spiegel, M., Lang, K.S., Akira, S., Thiel, V., and Ludewig, B. (2007). Control of coronavirus infection through plasmacytoid dendritic-cell-derived type I interferon. Blood *109*, 1131–1137.

Chang, C.H., Qiu, J., O'Sullivan, D., Buck, M.D., Noguchi, T., Curtis, J.D., Chen, Q., Gindin, M., Gubin, M.M., van der Windt, G.J.W., et al. (2015). Metabolic competition in the tumor microenvironment is a driver of cancer progression. Cell *162*, 1229–1241.

Cheng, P.N.-M., Lam, T.-L., Lam, W.-M., Tsui, S.-M., Cheng, A.W.-M., Lo, W.-H., and Leung, Y.-C. (2007). Pegylated recombinant human arginase (rhArg-peg5,000mw) inhibits the in vitro and in vivo proliferation of human hepatocellular carcinoma through arginine depletion. Cancer Res. *67*, 309–317.

Crispe, I.N. (2016). Hepatocytes as immunological agents. J. Immunol. *196*, 17–21.

Dantzer, R., O'Connor, J.C., Freund, G.G., Johnson, R.W., and Kelley, K.W. (2008). From inflammation to sickness and depression: when the immune system subjugates the brain. Nat. Rev. Neurosci. *9*, 46–56.

De Santo, C., Cheng, P., Beggs, A., Egan, S., Bessudo, A., and Mussai, F. (2018). Metabolic therapy with PEG-arginase induces a sustained complete remission in immunotherapy-resistant melanoma. J. Hematol. Oncol. *11*, 68.

Diez-Fernandez, C., Wellauer, O., Gemperle, C., Rüfenacht, V., Fingerhut, R., and Häberle, J. (2016). Kinetic mutations in argininosuccinate synthetase deficiency: characterisation and in vitro correction by substrate supplementation. J. Med. Genet. *53*, 710–719.

Dumas, M.-E., Kinross, J., and Nicholson, J.K. (2014). Metabolic phenotyping and systems biology approaches to understanding metabolic syndrome and fatty liver disease. Gastroenterology *146*, 46–62.

Feun, L.G., Marini, A., Walker, G., Elgart, G., Moffat, F., Rodgers, S.E., Wu, C.J., You, M., Wangpaichitr, M., Kuo, M.T., et al. (2012). Negative argininosuccinate synthetase expression in melanoma tumours may predict clinical benefit from arginine-depleting therapy with pegylated arginine deiminase. Br. J. Cancer *106*, 1481–1485.

Gabrilovich, D.I. (2017). Myeloid-derived suppressor cells. Cancer Immunol. Res. *5*, 3–8.

Geiger, R., Rieckmann, J.C., Wolf, T., Basso, C., Feng, Y., Fuhrer, T., Kogadeeva, M., Picotti, P., Meissner, F., Mann, M., et al. (2016). L-arginine modulates T cell metabolism and enhances survival and anti-tumor activity. Cell *167*, 829–842.e13.

Guerreiro, J.R., Lameu, C., Oliveira, E.F., Klitzke, C.F., Melo, R.L., Linares, E., Augusto, O., Fox, J.W., Lebrun, I., Serrano, S.M.T., and Camargo, A.C. (2009). Argininosuccinate synthetase is a functional target for a snake venom anti-hypertensive peptide: role in arginine and nitric oxide production. J. Biol. Chem. *284*, 20022–20033.

Guidotti, L.G., Borrow, P., Brown, A., McClary, H., Koch, R., and Chisari, F.V. (1999). Noncytopathic clearance of lymphocytic choriomeningitis virus from the hepatocyte. J. Exp. Med. *189*, 1555–1564.

Hafemeister, C., and Satija, R. (2019). Normalization and variance stabilization of single-cell RNA-seq data using regularized negative binomial regression. bioRxiv. https://doi.org/10.1101/576827.

Halpern, K.B., Shenhav, R., Matcovitch-Natan, O., Tóth, B., Lemze, D., Golan, M., Massasa, E.E., Baydatch, S., Landen, S., Moor, A.E., et al. (2017). Single-cell spatial reconstruction reveals global division of labour in the mammalian liver. Nature *542*, 352–356.

Hotamisligil, G.S. (2017). Inflammation, metaflammation and immunometabolic disorders. Nature *542*, 177–185.

Jenne, C.N., and Kubes, P. (2013). Immune surveillance by the liver. Nat. Immunol. *14*, 996–1006.

Jha, A.K., Huang, S.C.C., Sergushichev, A., Lampropoulou, V., Ivanova, Y., Loginicheva, E., Chmielewski, K., Stewart, K.M., Ashall, J., Everts, B., et al. (2015). Network integration of parallel metabolic and transcriptional data reveals metabolic modules that regulate macrophage polarization. Immunity *42*, 419–430.

Johnson, M.O., Wolf, M.M., Madden, M.Z., Andrejeva, G., Sugiura, A., Contreras, D.C., Maseda, D., Liberti, M.V., Paz, K., Kishton, R.J., et al. (2018). Distinct regulation of Th17 and Th1 cell differentiation by glutaminase-dependent metabolism. Cell *175*, 1780–1795.e19.

Kamphuis, E., Junt, T., Waibler, Z., Forster, R., and Kalinke, U. (2006). Type I interferons directly regulate lymphocyte recirculation and cause transient blood lymphopenia. Blood *108*, 3253–3261.

Kanehisa, M., and Goto, S. (2000). KEGG: kyoto encyclopedia of genes and genomes. Nucleic Acids Res. *28*, 27–30.

Kosack, L., Gawish, R., Lercher, A., Vilagos, B., Hladik, A., Lakovits, K., Bhattacharya, A., Schliehe, C., Mesteri, I., Knapp, S., and Bergthaler, A. (2017). The lipid-sensor TREM2 aggravates disease in a model of LCMV-induced hepatitis. Sci. Rep. *7*, 11289.

Kotas, M.E., and Medzhitov, R. (2015). Homeostasis, inflammation, and disease susceptibility. Cell *160*, 816–827.

Krzywinski, M., Schein, J., Birol, I., Connors, J., Gascoyne, R., Horsman, D., Jones, S.J., and Marra, M.A. (2009). Circos: an information aesthetic for comparative genomics. Genome Res. *19*, 1639–1645.

Lalazar, G., and Ilan, Y. (2014). Viral diseases of the liver. In Liver Immunology, M.E. Gershwin, J.M. Vierling, and M.P. Manns, eds. (Springer International Publishing), pp. 159–171.

Lam, T.L., Wong, G.K.Y., Chow, H.Y., Chong, H.C., Chow, T.L., Kwok, S.Y., Cheng, P.N.M., Wheatley, D.N., Lo, W.H., and Leung, Y.C. (2011). Recombinant human arginase inhibits the in vitro and in vivo proliferation of human melanoma by inducing cell cycle arrest and apoptosis. Pigment Cell Melanoma Res. *24*, 366–376.

Law, C.W., Chen, Y., Shi, W., and Smyth, G.K. (2014). voom: precision weights unlock linear model analysis tools for RNA-seq read counts. Genome Biol. *15*, R29.

Lee, J.S., Adler, L., Karathia, H., Carmel, N., Rabinovich, S., Auslander, N., Keshet, R., Stettner, N., Silberman, A., Agemy, L., et al. (2018). Urea cycle dysregulation generates clinically relevant genomic and biochemical signatures. Cell *174*, 1559–1570.e22.

Li, H., Wang, L., Yan, X., Liu, Q., Yu, C., Wei, H., Li, Y., Zhang, X., He, F., and Jiang, Y. (2011). A proton nuclear magnetic resonance metabonomics approach for biomarker discovery in nonalcoholic fatty liver disease. J. Proteome Res. *10*, 2797–2806.

Li, L., Mao, Y., Zhao, L., Li, L., Wu, J., Zhao, M., Du, W., Yu, L., and Jiang, P. (2019). p53 regulation of ammonia metabolism through urea cycle controls polyamine biosynthesis. Nature *567*, 253–256.

Liao, Y., Smyth, G.K., and Shi, W. (2014). featureCounts: an efficient general purpose program for assigning sequence reads to genomic features. Bioinformatics *30*, 923–930.

Ma, E.H., Bantug, G., Griss, T., Condotta, S., Johnson, R.M., Samborska, B., Mainolfi, N., Suri, V., Guak, H., Balmer, M.L., et al. (2017). Serine is an essential metabolite for effector T cell expansion. Cell Metab. *25*, 345–357.

Manza, L.L., Stamer, S.L., Ham, A.J., Codreanu, S.G., and Liebler, D.C. (2005). Sample preparation and digestion for proteomic analyses using spin filters. Proteomics *5*, 1742–1745.

McNab, F., Mayer-Barber, K., Sher, A., Wack, A., and O'Garra, A. (2015). Type I interferons in infectious disease. Nat. Rev. Immunol. *15*, 87–103.

Medzhitov, R. (2008). Origin and physiological roles of inflammation. Nature *454*, 428–435.

Meijer, A.J., Lamers, W.H., and Chamuleau, R.A. (1990). Nitrogen metabolism and ornithine cycle function. Physiol. Rev. *70*, 701–748.

Metzger, D., Li, M., and Chambon, P. (2005). Targeted somatic mutagenesis in the mouse epidermis. Methods Mol. Biol. *289*, 329–340.

Miyajima, M., Zhang, B., Sugiura, Y., Sonomura, K., Guerrini, M.M., Tsutsui, Y., Maruya, M., Vogelzang, A., Chamoto, K., Honda, K., et al. (2017). Metabolic shift induced by systemic activation of T cells in PD-1-deficient mice perturbs brain monoamines and emotional behavior. Nat. Immunol. *18*, 1342–1352.

Mostafavi, S., Yoshida, H., Moodley, D., LeBoité, H., Rothamel, K., Raj, T., Ye, C.J., Chevrier, N., Zhang, S.Y., Feng, T., et al.; Immunological Genome Project Consortium (2016). Parsing the interferon transcriptional network and its disease associations. Cell *164*, 564–578.

Mounce, B.C., Cesaro, T., Moratorio, G., Hooikaas, P.J., Yakovleva, A., Werneke, S.W., Smith, E.C., Poirier, E.Z., Simon-Loriere, E., Prot, M., et al. (2016). Inhibition of polyamine biosynthesis is a broad-spectrum strategy against RNA viruses. J. Virol. *90*, 9683–9692.

Muller, U., Steinhoff, U., Reis, L.F., Hemmi, S., Pavlovic, J., Zinkernagel, R.M., and Aguet, M. (1994). Functional role of type I and type II interferons in antiviral defense. Science *264*, 1918–1921.

Murray, P.J. (2016). Amino acid auxotrophy as a system of immunological control nodes. Nat. Immunol. *17*, 132–139.

Norata, G.D., Caligiuri, G., Chavakis, T., Matarese, G., Netea, M.G., Nicoletti, A., O'Neill, L.A.J., and Marelli-Berg, F.M. (2015). The cellular and molecular basis of translational immunometabolism. Immunity *43*, 421–434.

Olsen, J.V., de Godoy, L.M., Li, G., Macek, B., Mortensen, P., Pesch, R., Makarov, A., Lange, O., Horning, S., and Mann, M. (2005). Parts per million mass accuracy on an Orbitrap mass spectrometer via lock mass injection into a C-trap. Mol Cell Proteomics *4*, 2010–2021.

O'Neill, L.A.J., and Pearce, E.J. (2016). Immunometabolism governs dendritic cell and macrophage function. J. Exp. Med. *213*, 15–23.

Okin, D., and Medzhitov, R. (2012). Evolution of inflammatory diseases. Curr. Biol. *22*, R733–R740.

Pallett, L.J., Gill, U.S., Quaglia, A., Sinclair, L.V., Jover-Cobos, M., Schurich, A., Singh, K.P., Thomas, N., Das, A., Chen, A., et al. (2015). Metabolic regulation of hepatitis B immunopathology by myeloid-derived suppressor cells. Nat. Med. *21*, 591–600.

Pantel, A., Teixeira, A., Haddad, E., Wood, E.G., Steinman, R.M., and Longhi, M.P. (2014). Direct type I IFN but not MDA5/TLR3 activation of dendritic cells is required for maturation and metabolic shift to glycolysis after poly IC stimulation. PLoS Biol. *12*, e1001759.

Pearce, E.L., and Pearce, E.J. (2013). Metabolic pathways in immune cell activation and quiescence. Immunity *38*, 633–643.

Pearce, E.L., Walsh, M.C., Cejas, P.J., Harms, G.M., Shen, H., Wang, L.-S., Jones, R.G., and Choi, Y. (2009). Enhancing CD8 T-cell memory by modulating fatty acid metabolism. Nature *460*, 103–107.

Poillet-Perez, L., Xie, X., Zhan, L., Yang, Y., Sharp, D.W., Hu, Z.S., Su, X., Maganti, A., Jiang, C., Lu, W., et al. (2018). Autophagy maintains tumour growth through circulating arginine. Nature *563*, 569–573.

Protzer, U., Maini, M.K., and Knolle, P.A. (2012). Living in the liver: hepatic infections. Nat. Rev. Immunol. *12*, 201–213.

Rabinovich, S., Adler, L., Yizhak, K., Sarver, A., Silberman, A., Agron, S., Stettner, N., Sun, Q., Brandis, A., Helbling, D., et al. (2015). Diversion of aspartate in ASS1-deficient tumours fosters de novo pyrimidine synthesis. Nature *527*, 379–383.

Racanelli, V., and Rehermann, B. (2006). The liver as an immunological organ. Hepatology *43* (2, Suppl 1), S54–S62.

Rehermann, B., and Nascimbeni, M. (2005). Immunology of hepatitis B virus and hepatitis C virus infection. Nat. Rev. Immunol. *5*, 215–229.

Sanchez, K.K., Chen, G.Y., Schieber, A.M.P., Redford, S.E., Shokhirev, M.N., Leblanc, M., Lee, Y.M., and Ayres, J.S. (2018). Cooperative metabolic adaptations in the host can favor asymptomatic infection and select for attenuated virulence in an enteric pathogen. Cell *175*, 146–158.e15.

Schoggins, J.W., Wilson, S.J., Panis, M., Murphy, M.Y., Jones, C.T., Bieniasz, P., and Rice, C.M. (2011). A diverse range of gene products are effectors of the type I interferon antiviral response. Nature *472*, 481–485.

Schuler, M., Dierich, A., Chambon, P., and Metzger, D. (2004). Efficient temporally controlled targeted somatic mutagenesis in hepatocytes of the mouse. Genesis *39*, 167–172.

Sinclair, L.V., Rolf, J., Emslie, E., Shi, Y.B., Taylor, P.M., and Cantrell, D.A. (2013). Control of amino-acid transport by antigen receptors coordinates the metabolic reprogramming essential for T cell differentiation. Nat. Immunol. *14*, 500–508.

Smyth, G.K. (2004). Linear models and empirical bayes methods for assessing differential expression in microarray experiments. Stat. Appl. Genet. Mol. Biol. *3*, Article3.

Soga, T., Sugimoto, M., Honma, M., Mori, M., Igarashi, K., Kashikura, K., Ikeda, S., Hirayama, A., Yamamoto, T., Yoshida, H., et al. (2011). Serum metabolomics reveals γ-glutamyl dipeptides as biomarkers for discrimination among different forms of liver disease. J. Hepatol. *55*, 896–905.

St John-Williams, L., Blach, C., Toledo, J.B., Rotroff, D.M., Kim, S., Klavins, K., Baillie, R., Han, X., Mahmoudiandehkordi, S., Jack, J., et al.; Alzheimer's Disease Neuroimaging Initiative; Alzheimer's Disease Metabolomics Consortium (2017). Targeted metabolomics and medication classification data from participants in the ADNI1 cohort. Sci. Data *4*, 170140.

Stuart, T., Butler, A., Hoffman, P., Hafemeister, C., Papalexi, E., Mauck, W.M., 3rd, Hao, Y., Stoeckius, M., Smibert, P., and Satija, R. (2019). Comprehensive integration of single-cell data. Cell *177*, 1888–1902.e21.

Sturman, L.S., and Takemoto, K.K. (1972). Enhanced growth of a murine coronavirus in transformed mouse cells. Infect. Immun. *6*, 501–507.

Tranah, T.H., Vijay, G.K.M., Ryan, J.M., and Shawcross, D.L. (2013). Systemic inflammation and ammonia in hepatic encephalopathy. Metab. Brain Dis. *28*, 1–5.

Uhlén, M., Fagerberg, L., Hallström, B.M., Lindskog, C., Oksvold, P., Mardinoglu, A., Sivertsson, Å., Kampf, C., Sjöstedt, E., Asplund, A., et al. (2015). Tissue-based map of the human proteome. Science *347*, 1260419.

Van de Velde, L.A., and Murray, P.J. (2016). Proliferating helper T cells require rictor/mTORC2 complex to integrate signals from limiting environmental amino acids. J. Biol. Chem. *291*, 25815–25822.

van den Berghe, G. (1991). The role of the liver in metabolic homeostasis: implications for inborn errors of metabolism. J. Inherit. Metab. Dis. *14*, 407–420.

Virgin, H.W., Wherry, E.J., and Ahmed, R. (2009). Redefining chronic viral infection. Cell *138*, 30–50.

Wang, A., Luan, H.H., and Medzhitov, R. (2019). An evolutionary perspective on immunometabolism. Science *363*, 80.

Watford, M. (2003). The urea cycle: teaching intermediary metabolism in a physiological setting. Biochem. Mol. Biol. Educ. *31*, 289–297.

Wherry, E.J., and Kurachi, M. (2015). Molecular and cellular insights into T cell exhaustion. Nat. Rev. Immunol. *15*, 486–499.

Wisniewski, J.R., Zougman, A., Nagaraj, N., and Mann, M. (2009). Universal sample preparation method for proteome analysis. Nat Methods *6*, 359–362.

Wu, G., Bazer, F.W., Davis, T.A., Kim, S.W., Li, P., Marc Rhoads, J., Carey Satterfield, M., Smith, S.B., Spencer, T.E., and Yin, Y. (2009). Arginine metabolism and nutrition in growth, health and disease. Amino Acids *37*, 153–168.

Wu, D., Sanin, D.E., Everts, B., Chen, Q., Qiu, J., Buck, M.D., Patterson, A., Smith, A.M., Chang, C.-H., Liu, Z., et al. (2016). Type 1 interferons induce changes in core metabolism that are critical for immune function. Immunity *44*, 1325–1336.

York, A.G., Williams, K.J., Argus, J.P., Zhou, Q.D., Brar, G., Vergnes, L., Gray, E.E., Zhen, A., Wu, N.C., Yamada, D.H., et al. (2015). Limiting cholesterol biosynthetic flux spontaneously engages type I IFN signaling. Cell *163*, 1716–1729.

Zehn, D., and Wherry, E.J. (2015). Immune memory and exhaustion: clinically relevant lessons from the LCMV model. Adv. Exp. Med. Biol. *850*, 137–152.

Zhou, Z., Xu, M.J., and Gao, B. (2016). Hepatocytes: a key cell type for innate immunity. Cell. Mol. Immunol. *13*, 301–315.

Zinkernagel, R.M., Haenseler, E., Leist, T., Cerny, A., Hengartner, H., and Althage, A. (1986). T cell-mediated hepatitis in mice infected with lymphocytic choriomeningitis virus. Liver cell destruction by H-2 class I-restricted virus-specific cytotoxic T cells as a physiological correlate of the 51Cr-release assay? J. Exp. Med. *164*, 1075–1092.

STAR★METHODS

KEY RESOURCES TABLE

REAGENT or RESOURCE	SOURCE	IDENTIFIER
Antibodies		
anti-CD3e	BD Biosciences	Cat#553238
anti-CD28	BD Biosciences	Cat#553295
anti-CD8.2b:Pacific Blue, clone 53-5.8	Biolegend	Cat#140414
anti-CD4:FITC, clone H129.19	Biolegend	Cat#100408Cat#130308
anti-CD4:PE, clone GK1.5	Biolegend	Cat#100408
anti-CD3e:APC, clone 145-2C11	Biolegend	Cat#100312
anti-CD44:BV605, clone IM7	Biolegend	Cat#103047
anti-CD62L:AF700, clone MEL-14	Biolegend	Cat#104426
anti-CD19:APC-Cy7, clone 6D5	Biolegend	Cat#115530
anti-IFNγ: PE-Cy7, clone XMG1.2	Biolegend	Cat#505826
anti-IL-2: PE, clone JES6-5H4	Biolegend	Cat#503808
anti-TNFα: APC, clone MP6-XT22	Biolegend	Cat#506308
anti-STAT1	Cell Signaling Technology	Cat#39172
anti-ASS1	Abcam	Cat#ab170952
anti-OTC	Santa Cruz	Cat#sc-5157791
anti-CD16/32, clone: 93	Biolegend	Cat#101330
anti-CD16/32, clone: 93	Biolegend	Cat#101330
anti-CD16/32, clone: 93	Biolegend	Cat#101330
rat anti-IFN-α	PBL Interferon Source	Cat#22100-1
rabbit anti-IFN-α	PBL Interferon Source	Cat#32100-1
anti-rabbit HRP antibody	Jackson ImmunoReserach	Cat#711-036-152
Bacterial and Virus Strains		
MHV strain A59	Cervantes-Barragan et al., 2007	NA
LCMV strain Cl13	Bhattacharya et al., 2015	NA
Chemicals, Peptides, and Recombinant Proteins		
Polyinosine-olycytidylic acid (poly(I:C))	invivogen	Cat#tlrl-pic
Recombinant murine IFNβ	PBL Assay Science	Cat#12405-1
Recombinant murin IL-2	Thermo Fisher Scientific	Cat#34-8021-82
Liberase TM	Sigma	Cat#5401127001
Tamoxifen	Sigma	Cat#T5658
Sunflower oil	Sigma	Cat#S5007
α-Methyl-DL-aspartic acid	Sigma	Cat#M6001
L-Citrulline	Sigma	Cat#C7629
L-Arginine	Sigma	Cat#A5006
L-Ornithine	Sigma	Cat#O2375
β-mercaptoethanol	Sigma	Cat#M6250
$^{13}C_6$ arginine	Cambridge Isotope Laboratories	Cat#CLM-2265-H-PK
$^{13}C_5$ ornithine	Cambridge Isotope Laboratories	Cat#CLM-4724-PK
$^{13}C_4$ aspartic acid	Cambridge Isotope Laboratories	Cat#CLM-1801-H-PK
Carbamoyl phosphate	Sigma	Cat#C4135
Phosphatase inhibitor cocktail	Thermo Fisher Scientific	Cat#78447
Adenosine triphosphate	Thermo Fisher Scientific	Cat#R1441
QIAzol lysis reagent	QIAGEN	Cat#79306
Roti histofix	Carl Roth	Cat#P087.4

(*Continued on next page*)

Continued

REAGENT or RESOURCE	SOURCE	IDENTIFIER
Target retrieval solution	Dako	Cat#S1699
Taqman fast universal PCR mastermix	Thermo Fisher Scientific	Cat#4352042
Agilent Seahorse XF base medium	Agilent	Cat#102353-100
Red blood cell lysis buffer	eBioscience	Cat#00-430054
RPMI 1640 medium for SILAC	Thermo Fisher Scientific	Cat#88365
Dialyzed FCS	Thermo Fisher Scientific	Cat#A3382001
Heparin-Natrium-5000-ratiopharm	Ratiopharm	NA
Xylapan	Vetoquinol	NA
Narketan	Vetoquinol	NA
GP33-PE	NIH tetramer core facility	NA
NP396-APC	NIH tetramer core facility	NA
Protein transport inhibitor cocktail	eBioscience	Cat#00-4980-03
Cell stimulation cocktail	eBioscience	Cat#00-4970-93
Saponin	Sigma	Cat#47036
LCMV gp33-41 peptide (KAVYNFATC)	Peptide 2.0 Inc.	custom synthesis
LCMV np396-404 peptide (FQPQNGAFI)	Peptide 2.0 Inc.	custom synthesis
LCMV gp276-286 peptide (SGVENPGGYCL)	Peptide 2.0 Inc.	custom synthesis
LCMV gp64-80 peptide (GPDIYKGVYQFKSVEFD)	Peptide 2.0 Inc.	custom synthesis
Pegylated recombinant human ARG1	Bio-Cancer Treatment International Ltd.	Cat#PEG-BCT-100
Recombinant murine IFNα	PBL Interferon Source	Cat#12100-1
TMB Solution	Thermo Fisher Scientific	Cat#002023
Critical Commercial Assays		
Agilent Seahorse XF Cell Mito Stress Test Kit	Agilent	Cat#10315-100
CD8a+ T Cell Isolation Kit	Miltenyi Biotec	Cat#130-104-075
Absolute IDQ P180 Kit	Biocrates Life Sciences AG	NA
Precellys CK14 Lysing Kit	VWR	Cat#432-3751
RevertAid First Strand cDNA Synthesis Kit	Thermo Fisher Scientific	Cat#K1612
Deposited Data		
Proteomic data	This study	PRIDE: PXD011122
Transcriptomic data (superfamily)	This study	GEO: GSE123688
Experimental Models: Organisms/Strains		
Mouse:Cre-Alb ERT2	Schuler et al., 2004	NA
Mouse:Ifnar1$^{-/-}$	Muller et al., 1994	NA
Mouse:Ifnar1$^{fl/fl}$	Kamphuis et al., 2006	NA
Oligonucleotides		
Ef1α Taqman primers and probes	Bhattacharya et al., 2015	NA
Otc Taqman Gene Expression Assay	Thermo Fisher Scientific	Cat#Mm00493267_m1
Ass1 Taqman Gene Expression Assay	Thermo Fisher Scientific	Cat#Mm00711256_m1
LCMV *NP* Taqman primers and probes	Bhattacharya et al., 2015	NA
Software and Algorithms		
FlowJo v8.7	FlowJo	FlowJo
Trace Finder v4.1	Thermo Fisher Scientific	Trace Finder
Cell Ranger v3.0.2	10X Genomics	CellRanger
MetIDQ, Version 5-4-8-DB100-Boron-2607	Biocrates Life Sciences AG	MetIDQ
HistoQuest TM software	TissueGnostics GmbH	HistoQuest
Xcalibur v2.1	Thermo Fisher Scientific	Xcalibur
ExpressCluster v1.3	Harvard Medical School	ExpressCluster
ClueGO v2.3.3	Bindea et al., 2009	Cytoscape ClueGO

(*Continued on next page*)

Continued		
REAGENT or RESOURCE	SOURCE	IDENTIFIER
TopHat2 v2.0.10	John's Hopkins University	TopHat
Cufflinks/Cuffdif/Cuffmerge v2.2.1	Cole Trapnell Lab	Cufflinds/Cuffdiff
featureCounts	Walter and Eliza Hall Insitute of Medical Research	Subread Package
Seurat v3.1.0	Satija Lab, New York Genome Center	Satija Lab
R	Cran	R-Project
limma R package	Bioconductor	limma R package
voom limma function	voom	voom
ProteoWizard Library v2.1.2708	ProteoWizard	ProteoWizard
MASCOT v2.3.02	MatrixScience	MatrixScience
Phenyx v2.5.14	GeneBio	GeneBio
Isobar Software	Breitwieser and Colinge, 2013	NA
Prism v7-8	Graphpad Software	Graphpad Software
Wave Desktop v2.0	Agilent	Wave Desktop

LEAD CONTACT AND MATERIALS AVAILABILITY

Inquires for further information or requests for resources and reagents should be directed and will be fulfilled by the lead contact Andreas Bergthaler (abergthaler@cemm.oeaw.ac.at). This study did not generate new unique reagents.

EXPERIMENTAL MODEL AND SUBJECT DETAILS

Mice

C57BL/6J mice were originally obtained from The Jackson Laboratory, *Ifnar1*$^{fl/fl}$ mice from Ulrich Kalinke (TWINCORE, Centre for Experimental and Clinical Infectiton Research, Hannover, Germany) and Cre-Alb ERT2 mice from Pierre Chambon (Institut de Génétique et de Biologie Moléculaire et Cellulaire, Illkirch, France). Mice were bred and maintained under specific pathogen-free (SPF) conditions at the Institute for Molecular Biotechnology of the Austrian Academy of Sciences, Vienna, Austria. Animal experiments were conducted in individual ventilated cages according to the respective animal experiment licenses (BMWFW-66.009/0199-WF/V/3v/2015 and BMWFW-66.009/0361-WF/V/3b/2017) approved by the institutional ethical committees and the institutional guidelines at the Department for Biomedical Research of the Medical University of Vienna. Mice were 8 to 12 weeks old and age- and sex-matched within experiments. Male and female mice were used interchangeably between experiments and no striking sex-differences were observed. For experiments using murine hepatitis virus (MHV), mice were maintained under SPF conditions at the Kantonsspital St. Gallen Medical Research Center, St. Gallen Switzerland. MHV experiments were conducted in individually ventilated cages according to the animal experiment license (SG/14/18.30861) approved by federal and cantonal ethical committees.

Viruses and cell lines

Lymphocytic choriomeningitis virus (LCMV) was grown on BHK-21 cells (baby hamster kidney cells derived from 5 unsexed newborns, ATCC CCL-10) and titer was determined in a modified focus forming assay using Vero cells (female green monkey kidney cells, ATCC CCL-81) (Bhattacharya et al., 2015). Mice were intravenously infected with 2x10^6 focus forming units (FFU) of LCMV.

Murine hepatitis virus strain A59 (MHV) was generated using 17CL1 cells (spontaneously transformed 3T3 cell line established from unsexed BALB/c mouse embryos) (Sturman and Takemoto, 1972) and titer was determined by standards plaque assay on L929 cells (male murine fibroblasts, ATCC CCL-1) (Cervantes-Barragan et al., 2007). Mice were intraperitoneally infected with 10^3 plaque forming units (PFU) of MHV.

METHOD DETAILS

Infections

Mice were sacrificed at the time points indicated in the legends. Tissue samples were snap frozen in liquid nitrogen and stored at -80°C until further analyses. For serum collection, blood was collected at the respective time points, samples were centrifuged at 10.000 rpm for 5 min at 4°C. The serum was transferred into a new tube and stored at -80°C until further analyses.

Pharmacological perturbations

Pegylated recombinant human Arginase 1 (PEG-BCT-100, Bio-Cancer Treatment International) was administered to mice via intraperitoneal injection at a dosage of 50 mg/kg. Mice were treated twice per week. Treatment was started one day prior to LCMV infection. Control mice received the same volume of PBS.

Conditional Ifnar1 ablation on hepatocytes

Tamoxifen (T5658, Sigma) was dissolved in sterile sunflower oil (S5007, Sigma) containing 10% ethanol (v/v) and stored at −20°C for a maximum of 2 weeks. To induce hepatocyte specific *Ifnar1* ablation, Cre-Alb ERT2 *Ifnar1*$^{fl/fl}$ and Cre-Alb ERT2 *Ifnar1*$^{+/+}$ control mice were administered 50 mg/kg tamoxifen intraperitoneally for 5 consecutive days (Metzger et al., 2005). Experiments were started the day after the last dose.

poly(I:C) treatment

Mice were challenged with poly(I:C) (invivogen, #tlrl-pic) via intraperitoneal injection at a dosage of 4 mg/kg. Control mice received PBS. Liver tissue was harvested and analyzed via real-time PCR at the indicated time points.

Isolation of primary murine hepatocytes

Mice were anesthetized (Ketamine/Xylazine: 1:3, 0.1 ml/10 g mouse, Vetoquinol) and the liver was cannulated via the portal vein and perfused with 20 mL HBSS (GIBCO) containing 0.5 mM EGTA (Sigma) followed by digestion of the liver with 20 mL L15 medium (GIBCO) containing 40 mg/L Liberase (Roche) at a rate of 5 mL/min. The liver was isolated, placed in a Petri dish with digestion medium (L15 with 40 mg/L Liberase) and the liver capsule was diligently removed. Cells were centrifuged at 50 g for 5 min at 4°C, resuspended in William's E medium containing 10% FCS (PAA) and 1% Penicillin-Streptomycin-Glutamine (Thermo Fisher Scientific) and plated. Cells were stimulated at the time of plating.

Metabolite tracing

Naive or LCMV-infected C57BL/6J mice were given an intravenous bolus of 500 μg $^{13}C_6$ labeled arginine (Cambridge Isotope Laboratories, CLM-2265-H-PK) dissolved in PBS. Mice were sacrificed 20 min afterward and serum and tissues of interest were harvested and stored at −80°C.

Per mg tissue (approximately 100 mg per sample), 3 μL 80% (v/v) methanol were added and tissue samples were homogenized using a Precellys 24 tissue homogenizer (Precellys CK14 lysing kit, Bertin). 50 μL of homogenized tissue or serum were mixed with 450 μL of methanol and 250 μL of water and vortexed for 10 s. Afterward, 450 μL chloroform were added per sample and mixed by vortexing for 10 s, incubated on ice for 5 min and vortexed again for 10 s before centrifuging them for 10 min at 1000 g. The upper aqueous phase was collected, dried (nitrogen evaporator) and reconstituted in 50 μL of methanol. Samples were centrifuged for 10 min at 1000 g and supernatants were used for LC-MS analysis.

A Vanquish UHPLC system (Thermo Scientific) coupled to an Orbitrap Fusion Lumos (Thermo Scientific) mass spectrometer was used for the LC-MS analysis. The chromatographic separation for samples was carried out on an ACQUITY UPLC BEH Amide, 1.7 μm, 2.1x100 mm analytical column (Waters) equipped with a VanGuard: BEH C18, 2.1x5mm pre-column (Waters). The column was maintained at a temperature of 40°C and 2 μL sample were injected per run. The mobile phase A was 0.15% v/v formic acid in water and mobile phase B was 0.15% v/v formic acid in 85% v/v acetonitrile with 10 mM ammonium formate. The gradient elution with a flow rate 0.4 mL/min was performed with a total analysis time of 17 min. The mass spectrometer was operated in a positive electrospray ionization mode: spray voltage 3.5 kV; sheath gas flow rate 60 arb; auxiliary gas flow rate 20 arb; capillary temperature 285°C. For analysis, a full MS scan mode with a scan range m/z 50 to 400, resolution 120.000, AGC target 2e5 and a maximum injection time 50 ms was applied. For detection of urea the scan range was adjusted to m/z 50 to 250 at a resolution of 500.000.

Enzymatic assays

Liver tissue lysis and buffer conditions for enzymatic assays for OTC and ASS1 were previously described (Diez-Fernandez et al., 2016; Guerreiro et al., 2009). Briefly, liver tissue was lysed in 10 mM HEPES, 0.05% Triton X-100, 0.5 mM DTT, 2 mM EDTA, 1X Halt Protease and Phosphatase Inhibitor Cocktail (Thermo Fisher Scientific, 78447), pH 7.4 using a TissueLyser (QIAGEN) at 30 Hz for 3x 30 s. Protein concentrations were measured in the cleared lysate using a Bradford assay. 100 μg of protein were used for each assay. For OTC activity, 200 μL of 50 mM Tris-Acetate, 2 mM EDTA, 1 mM L-Ornithine $^{13}C_5$ (Cambridge Isotype Laboratories, CLM-4724-PK, 1 mM Carbamoyl Phosphate (Sigma, C4135), 1X Halt Protease and Phosphatase Inhibitor Cocktail (Thermo Fisher Scientific, 78447), pH 8.3 and samples were incubated for 30 min at 37°C. For ASS1 activity, 200 μL of 20 mM HEPES, 2 mM ATP (Thermo Fisher Scientific, R1441), 5 mM $MgCl_2$, 1 mM L-Aspartate $^{13}C_4$ (Cambridge Isotype Laboratories, CLM-1801-H-PK), 1 mM L-Citrulline (Sigma, C7629), 1X Halt Protease and Phosphatase Inhibitor Cocktail (Thermo Fisher Scientific, 78447), pH 7 and samples were incubated for 30 min at 37°C. For experiments using the ASS1-specific inhibitor α-Methyl-DL-aspartic acid (MDLA, M6001, Sigma), 100 μg of total liver protein extract were treated with 10 mM MDLA and the enzymatic assay was performed as described above (Guerreiro et al., 2009).

Following the incubation, 10 μL of sample was immediately mixed with 90 μL methanol and subsequently prepared for mass spectrometric analysis of labeled downstream metabolites.

^{13}C labeled argininosuccinate or ^{13}C labeled citrulline was detected using a Vanquish UHPLC system (Thermo Scientific) coupled to an Orbitrap Fusion Lumos (Thermo Scientific) mass spectrometer was used for the LC-MS analysis. The chromatographic separation for samples was carried out on an ACQUITY HSS T3, 1.8 μm, 2.1x100 mm analytical column (Waters) equipped with a VanGuard HSS T3, 2.1x5 mm pre-column (Waters). The column was maintained at a temperature of 40°C and 2 μL sample were injected per run. The mobile phase A was 0.1% formic acid (v/v) in water and mobile phase B was 0.1% formic acid (v/v) in methanol. The gradient elution with a flow rate 0.5 mL/min was performed with a total analysis time of 10 min. The mass spectrometer was

operated in a positive electrospray ionization mode: spray voltage 3.5 kV; sheath gas flow rate 60 arb; auxillary gas flow rate 20 arb; capillary temperature 285°C. For the analysis a full MS scan mode with a scan range m/z 80 to 400, resolution 500.000, AGC target 2e5 and a maximum injection time 50 ms was applied.

Gene expression analyses of sorted primary murine hepatocytes

Primary murine hepatocytes from naive or LCMV Cl13 infected female animals were isolated as described above. After isolation, cells derived from one liver were resuspended in 500 μL PBS containing anti-CD16/32 (1:200, clone: 93, Biolegend) and incubated for 10 min at room temperature. Next, immune cells (CD45.2: PE clone: 104, Biolegend, 1:200) and dead cells (Fixable Viability Dye: eFluor 780, eBioscience, 1:2000) were stained for 20 min at 4°C. Cells were washed with PBS and 50.000 to 200.000 hepatocytes (viable $CD45.2^{-}$, purity > 95%) were sorted into PBS containing 0.04% BSA on a Sony SH800 Cell Sorter using a 130 μm chip and used for gene expression analyses. For real-time PCR analyses, cells from each mouse (n = 3) were centrifuged at 1500 rpm for 5 min and resuspended in QIAzol for further analyses. For single cell RNA-seq, cells from 2-3 mice were pooled in equal amounts, centrifuged, and resuspended in PBS containing 0.04% BSA to obtain a concentration of 800.000 cells/mL. This cells suspension was subsequently used for droplet based single cell RNA-seq using a 10X Genomics Chromium Single Cell Controller and standard protocols. Samples were sequenced with an Illumina HiSeq 3000/4000 instrument with 3 samples multiplexed per lane and run on a 75bp paired-end flow cell.

Immunohistochemistry

Liver pieces were fixed in Roti Histofix (P087.4, Carl Roth) for 48h. Afterward the fixation solution was exchanged to 70% (v/v) ethanol and stored until paraffin-embedment. For immunohistochemical stainings 2 μm FFPE consecutive sections cut, heat-mediated antigen retrieval was performed in citrate buffer at pH 6.0 (S1699; Dako, Agilent, Santa Clara, CA, USA). Sections were stained with antibodies specific to STAT1 monoclonal rabbit (1:200; 9172; Cell Signaling Technology), ASS1 monoclonal rabbit (1:400; ab170952; Abcam), OTC monoclonal rabbit (1:500, sc-5157791; Santa Cruz), using standard protocols.

Cytokine determination

IFN-α serum levels were determined via enzyme-linked immunosorbent assay (ELISA) as described previously (Bhattacharya et al., 2015). In brief, microplates (675074, Greiner Bio-One) were coated with rat anti-mIFN-α capture antibody (22100-1, PBL Interferon Source, 1:54 dilution in PBS). Pre-diluted serum samples (1:10 to 1:20 in PBS) and mIFN-α standards (12100-1, PBL Interferon Source) were added and incubated in a humidified chamber overnight at 4°C. For detection, a primary rabbit anti-mIFN-α antibody (32100-1, PBL Interferon Source, 1:738 dilution in PBS), a secondary anti-rabbit HRP antibody (711-036-152, Jackson ImmunoResearch) and TMB solution (002023, Thermo Fisher Scientific) were used. The color reaction was read out on a plate reader at 450 nm.

Blood chemistry

Mouse serum was pre-diluted 1:8 in PBS and levels of alanine aminotransferase (ALT) and aspartate aminotransferase (AST) were spectrophotometrically analyzed using a Cobas C311 Analyzer (Roche). For blood ammonia, mice were terminally anesthetized (Ketamine/Xylazine: 1:3, 0.1 ml/10 g mouse, Vetoquinol) and blood was sampled via heart puncture. Blood was collected in MiniCollect blood EDTA tubes (Greiner Bio-One). Plasma was obtained by centrifugation of whole blood at 4.000 rpm for 10 min at 4°C and pre-diluted 1:1 in 0.9% (w/v) NaCl solution. Samples were kept on ice, in the dark and in closed tubes until analysis of blood ammonia on a Cobas 8000 analyzer (Roche).

RNA isolation and real-time PCR

Tissues were homogenized using a TissueLyser II (QIAGEN). Total RNA was extracted from homogenized liver tissue using QIAzol lysis reagent as per the manufacturer's instructions (79306, QIAGEN). Reverse transcription from RNA to cDNA was carried out using random primers and the First Strand cDNA Synthesis Kit (K1612, Thermo Fisher Scientific). Real-time PCR was performed with Taqman Fast Universal PCR Mastermix (4352042, Thermo Fisher Scientific) and Taqman Gene Expression Assays (Thermo Fisher Scientific) for *Otc* (Mm00493267_m1) and *Ass1* (Mm00711256_m1). Expression levels of LCMV NP (5′-CAAGTATTCACACGG CATGGA-3′, 5′-TGGGAGAGCACCTATAACTGATA-3′ and 5′-[6FAM]TGATCTCTTCAATGCACAGCCTGGGC[BHQ1]-3′) and *Ef1α* (5′-GCAAAAACGACCCACCAATG-3′, 5′-GGCCTTGGTTCAGGATA-3′, and 5′-[6FAM]CACCTGAGCAGTGAAGCCAG[TAM]-3′) were measured by corresponding probe and primer sets as described previously (Bhattacharya et al., 2015).

Metabolic flux measurements

Oxygen consumption rate (OCR) and extracellular acidification rate (ECAR) were determined on a Seahorse XFe96 Analyzer (Agilent) using the Seahorse XF Cell Mito Stress test kit (Agilent, 103015-100). 25.000 primary hepatocytes were plated per well respectively. In brief, cells were treated with selected stimuli for the indicated time points. Prior to measurement, media was changed to XF Base Medium (Agilent 102353-100) containing glucose (10 mM), sodium pyruvate (1 mM) and L-glutamine (2 mM) and cells were incubated

for 1h. The assay was run according to the manufacturer's instructions. Oligomycin (2 μM), Carbonyl cyanide-p-trifluoromethoxyphenylhydrazone (FCCP, 0.25 μM) and Rotenone/Antimycin A (500 nM) were subsequently injected into wells at the desired time points. Raw data were analyzed using Wave Desktop Software (Agilent, version 2.0) and exported and graphed in GraphPad Prism (GraphPad Sorftware, version 7.0a).

CD8 T cell isolation and *in vitro* activation

Spleens of mice were harvested and dissociated through a 40 μm cell strainer (Falcon). The pellet was resuspended in 1 mL red blood cell lysis buffer (eBioscience, #00-4300-54) and incubated at room temperature for 1 min. Subsequently the reaction was stopped by addition of 9 mL PBS. Cells were counted and CD8 T cells isolated using a magnetic activated cell sorting negative selection (MACS) kit according to the manufacturer's instructions (Miltenyi Biotec, #130-104-075). 96 well plates were coated over night at 4°C with 1 μg/mL anti CD3e (BD, #553238) and 2 μg/mL anti CD28 (BD, #553295) in a total volume of 60 μL PBS per well. Wells were washed with PBS and $5x10^4$ cells were plated per well in RPMI 1640 medium for SILAC (Thermo Fisher Scientific, #88365), supplemented with 10% dialyzed FCS (Thermo Fisher Scientific, # A3382001), 1% Penicillin-Streptomycin-Glutamine (Thermo Fisher Scientific), 50 μM β-mercaptoethanol (Sigma, #M6250) and 20 U/mL IL-2 (Thermo Fisher Scientific, #34-8021-82). Medium was supplemented with indicated concentrations of L-arginine (Sigma, #A5006) and/or L-ornithine (Sigma, #O2375) and cultured up to 72h before proceeding with intracellular cytokine staining.

Flow cytometry

To sample blood, 3-4 drops of blood were collected in 1 mL MEM medium (GIBCO) supplemented with 2000 U/L heparin (Heparin-Natrium-5000-ratiopharm). Red blood cells were lysed by adding 500 μL red blood cell lysis buffer (eBioscience, #00-4300-54) and incubated at room temperature for 1 min. Samples were spun down (1500 rpm), resuspended in 100 μL PBS and plated in a 96 well plate. Spleens were dissociated into a single cell suspension using a 40 μm cell strainer (Falcon) and resuspended in 10 mL of PBS (GIBCO). An aliquot of cell suspension was used for counting to calculate total number of cells per spleen. 200 μL per sample (approx. $2x10^6$ cells) were plated in a 96 well plate. Plates were spun and supernatants were removed.

For tetramer staining, cells were resuspended in 25 μL PBS containing GP33 (1:500) and NP396 (1:250) tetramers (NIH Tetramer Core Facility) and incubated at 37°C for 15 min. Next, 25 μL PBS containing anti-CD16/32 (Biolegend; 1:200, clone: 93) were added and incubated for 10 min at room temperature. 25 μL of a master mix of the desired surface marker antibodies (CD8.2b: Pacific Blue clone 53-5.8; CD4: FITC, clone H129.19; CD4: PE, clone GK1.5; CD3: APC, clone 145-2C11; CD44: BV605, clone IM7; CD62L: AF700, clone MEL-14; CD19: APC-Cy7, clone 6D5; all Biolegend; 1:200 in PBS) and Fixable Viability Dye eFluor 780 (eBioscience; 1:2000 in PBS) were added and cells were incubated for 20 min at 4°C. Cells were washed with FACS buffer (PBS, 2% FCS) and fixed in 4% Paraformaldehyde (Sigma) in PBS for 10 min. Subsequently samples were washed twice with FACS buffer, resuspended in 100 μL and analyzed by flow cytometry.

For intracellular cytokine staining (ICS), cell pellets were resuspended in 50 μL of RPMI 1640 medium (GIBCO) supplemented with 10% FCS (PAA) and 1% Penicillin-Streptomycin-Glutamine (Thermo Fisher Scientific), 50 μM β-mercaptoethanol (Sigma), containing LCMV peptides (1:1000, Peptide 2.0 Inc.) and Protein Transport Inhibitor Cocktail (eBioscience, #00-4980-03; 1:500, Thermo Fisher Scientific). As positive control, cells were treated with Cell Stimulation Cocktail (eBioscience, #00-4970-93). Cells were incubated for 4 h at 37°C and then surface antigens were stained as described above. Afterward, a master mix of desired antibodies in 25 μL FACS buffer containing 0.05% saponin (Sigma, 47036) against intracellular antigens of interest were added and incubated for 90 min at 4°C (IFNγ: PE-Cy7, clone XMG1.2; IL-2: PE, clone JES6-5H4; TNFα: APC, clone MP6-XT22; all Biolegend. all 1:200). Next, cells were washed twice with FACS buffer, resuspended in 100 μL and analyzed by flow cytometry.

Quantitative proteomics

Three TMT 6-plex runs were carried out to monitor the changes in liver protein abundance during the entire course of infection. The first run included biological replicates from day 2 and day 8 after infection along with the uninfected controls. The second run included replicate samples of uninfected controls, day 30 and day 60 after infection. To account for the effect of aging, the third run included uninfected controls at day 0 and day 123, along with infected samples from day 123 (all in biological replicates). Identical uninfected controls (day 0) were included in all three TMT 6-plex runs as an internal control to monitor reproducibility between the runs. Liver tissues were homogenized (TissueLyser II, QIAGEN) in 1.5 mL 50 mM HEPES buffer, pH 8.5 supplemented with 2% sodium dodecyl sulfate (SDS). The protein concentrations were determined by the bicinchoninic acid assay (BCA, Pierce Biotechnology, Thermo Scientific, IL). Further processing was adapted from a filter-aided sample preparation (FASP) method previously described (Manza et al., 2005; Wisniewski et al., 2009). For each sample, 100 μg of the liver tissue lysate was reduced with 100 mM dithiothreitol (DTT) and transferred into VIVACON 500 filter unit (Vivaproucts, Littleton, MA). SDS-containing buffer was removed from the sample by centrifugation and exchanged with 8 M urea in 100 mM Tris-HCl buffer. Proteins were alkylated with 50 mM iodoacetamide and washed with 50 mM triethyl ammonium bicarbonate (TEAB). Finally, porcine trypsin (Promega, Madison, WI) was used for protein digestion in an enzyme to protein ratio of 1:100 w/w.

For relative protein quantitation, six samples from each run were separately derivatized with TMT 6-plex reagents (ABI, Framingham, MA) according to the instructions provided by the manufacturer. The combination of the TMT 6-plex labels was as follows: (i) Run 1: day 0 uninfected (TMT 126 and 127), day 2 infected (TMT 128 and 129) and day 8 infected (TMT 130 and 131); (ii) Run 2: day 0 uninfected (126 and 127), day 30 infected (128 and 129) and day 60 infected (130 and 131); and (iii) Run 3: day 0 (126 and 127), day 123 uninfected (128 and 129) and day 123 infected (130 and 131).

The TMT-labeled tryptic digests were pooled and concentrated by solid phase extraction (SPE) (MacroSpin columns 30-300 μg capacity, The Nest Group, Southborough, MA, USA). Samples were treated with 20 mM ammonium formate prior to injection onto a Phenomenex column (150 × 2.0 mm Gemini®NX-C18 3 μm 110Å; Phenomenex, Torrance, CA, USA) in an Agilent 1200 series HPLC (Agilent Biotechnologies, Palo Alto, CA) with UV detection at 214 nm. HPLC solvent A consisted of 20 mM ammonium formate, pH 10 in 5% acetonitrile. Peptides were separated at flow rate of 100 μL/min and eluted from the column with a non-linear gradient ranging from 0 to 100% solvent B (20 mM ammonium formate, pH 10 in 90% acetonitrile). Seventy-two time-based fractions were collected, acidified, and pooled into 50 HPLC vials based on the UV trace. After removal of organic solvent in a vacuum centrifuge, samples were reconstituted to 10 μL with 5% formic acid (Bennett et al., 2011). Individual fractions were further analyzed at pH 2.4 by Agilent 1200 nano-HPLC system (Agilent Biotechnologies, Palo Alto, CA) coupled to a hybrid LTQ Orbitrap Velos mass spectrometer (ThermoFisher Scientific, Waltham, MA). Data were acquired utilizing the Xcalibur software version 2.1. Briefly, single fractions were loaded onto a trap column (Zorbax 300SB-C18 5 μm, 5 × 0.3 mm, Agilent Biotechnologies, Palo Alto, CA) with a binary pump at a flow rate of 45 μL/min. Solvents for LCMS separation were composed of 0.1% trifluoracetic acid (TFA) in water (solvent A) and 0.1% TFA in 70% methanol and 20% isopropanol (solvent B). The peptides were eluted by back-flushing from the trap column onto a 16 cm fused silica analytical column with an inner diameter of 50 μm packed with C18 reversed-phase material (ReproSil-Pur 120 C18-AQ, 3 μm, Dr. Maisch GmbH, Ammerbuch-Entringen, Germany). Elution was achieved with a 27 min gradient ranging from 3 to 30% solvent B, followed by a 25 min gradient from 30 to 70% solvent B and, finally, a 7 min gradient from 70 to 100% solvent B at a constant flow rate of 100 nL/min. The analysis was performed in a data-dependent acquisition mode. The 10 most intense ions were isolated and fragmented by high-energy collision-induced dissociation (HCD) for peptide identification and relative quantitation of TMT reporter ions. Dynamic exclusion for selected ions was 60 s and a single lock mass at *m/z* 445.120024 ($Si(CH3)_2O)6)_{20}$ (Olsen et al., 2005) was used for internal mass calibration with the target loss mass abundance of 0%. Maximal ion accumulation time allowed was 500 ms and overfilling of the C-trap was prevented by automatic gain control set to 10^6 ions for a full FTMS scan and 5×10^5 ions for MS^n HCD. Intact peptides were detected in the Orbitrap mass analyzer at resolution of 30,000 with the signal threshold of 2,000 counts for triggering an MSMS event. The maximum ion scan time was set to 200 ms for acquiring 1 microscan at a resolution of 7500.

Targeted LC-MS based metabolite measurements

Tissue samples were homogenized using a Precellys 24 tissue homogenizer (Precellys CK14 lysing kit, Bertin). Per mg tissue, 3 μL of 80% (v/v) methanol were added. 10 μL of homogenized tissue sample or serum were mixed with 10 μL of an isotopically labeled internal standard mixture in a hydrophobic 96 well filter plate. Aliquots of 300 μL of methanol were added and mixed for 20 min at 450 rpm. Afterward, the sample extracts were collected by centrifuging the filter plate for 5 min at 500 g. A Vanquish UHPLC system (Thermo Scientific) coupled with an Orbitrap Q Exactive (Thermo Scientific) mass spectrometer was used for the LC-MS analysis. The chromatographic separation for samples was carried out on an ACQUITY UPLC BEH Amide, 1.7 μm, 2.1x100 mm analytical column (Waters) equipped with a VanGuard: BEH C18, 2.1x5mm pre-column (Waters). The column was maintained at a temperature of 40°C and 2 μL sample were injected per run. The mobile phase A was 0.15% formic acid (v/v) in water and mobile phase B was 0.15% formic acid (v/v) in 85% acetonitrile (v/v) with 10 mM ammonium formate. The gradient elution with a flow rate 0.4 mL/min was performed with a total analysis time of 17 min. The Orbitrap Q Exactive (Thermo Scientific) mass spectrometer was operated in an electrospray ionization positive mode, spray voltage 3.5 kV, aux gas heater temperature 400°C, capillary temperature 350°C, aux gas flow rate 12. The metabolites of interest were analyzed using a full MS scan mode, scan range m/z 50 to 400, resolution 35000, AGC target 1e6, maximum IT 50ms. The Trace Finder 4.1 software (Thermo Scientific) was used for the data processing. Seven-point linear calibration curves with internal standardization and 1/x weighing was constructed for the quantification of metabolites.

Metabolite measurements using the AbsoluteIDQ p180 Kit (Biocrates Life Science AG) were performed as described previously (St. John-Williams et al., 2017). In brief, per mg of tissue, 6 μL ethanol/phosphate buffer (85:15 v/v ethanol/10 mM phosphate buffer) were added and the tissue was homogenized (TissueLyser II, QIAGEN). Samples were centrifuged at 5,000 g for 5 min at 4°C and the supernatants were transferred to a fresh tube and used for analyses. For serum samples, blood was collected from mice in blood collection tubes and centrifuged at 12,000 g for 5 min to obtain sera. The serum was transferred into a fresh tube and stored at −80°C until analyses. The samples were analyzed on a Xevo TQ-MS (Waters) mass spectrometer using an Acquity UHPLC (Waters) system, operated with MassLynx V4.1 (Waters). Samples and additional blanks, calibration standards measurements, quality controls, and analyses were prepared according to the user manual.

QUANTIFICATION AND STATISTICAL ANALYSIS

RNA-seq data processing

RNA quality and integrity were assessed via an Experion RNA HighSense chip (Biorad). The library for RNA-seq was prepared using the TruSeq RNA sample preparation kit v2 (Illumina) according to the manufacturer's protocol. Quality control analysis was performed on all samples of the cDNA library by Experion DNA Analysis chip (Biorad) and Qubit Fluorometric quantitation (Life Tech).

7 or up to 17 samples were multiplexed per lane and run on a 50bp single-end flow cell in a HiSeq2000 or HiSeq3000 sequencer (Illumina), respectively. Called bases by the Illumina Realtime Analysis software were converted into BAM format using Illumina2bam and demultiplexed using BamIndexDecoder (https://github.com/wtsi-npg/illumina2bam). The RNA-seq analysis pipeline was performed with Tuxedo. Reads were mapped on the mouse reference genome (*Mus musculus*, Ensembl e87, December 2016) using TopHat2 (v2.0.10). Cufflinks (v2.2.1) was employed to assemble transcripts from spliced read alignments, using the Ensembl e87 transcriptome as the reference as well as *de novo* assembly of transcript models. Further, differential analysis of gene expression was quantified with Cuffdiff (v2.2.1). Transcriptome sets of all replicates for each sample group were combined with Cuffmerge. Expression values in graphs are reported as FPKM (fragments per kilobase pf transcript per million). Differential gene expression is attested based on expression level $\geq$ 1 FPKM, adjusted p value $\leq$ 0.05 and absolute log2 fold-change of 1 (heatmap) or 0.6 (circos plot).

Single cell RNA-seq processing

We performed single-cell RNA-sequencing on a 10X Genomics Chromium Single Cell Controller with the Chromium Single Cell 3′ V3 Kit following the manufacturer's instructions. Sequencing was performed on an Illumina HiSeq 3000 instrument in 2x75bp paired-end mode. We used the Cell Ranger (v3.0.2) 10X Genomics software to demultiplex the raw sequencing data and align them to the mouse GRCm38 reference genome. We proceeded with the analysis of the UMI counts using the R Bioconductor package Seurat v3.1.0 (Stuart et al., 2019). The two samples, uninfected and 2 days post infection, were merged and processed together. Cells with more than 30% mitochondrial content were discarded. Mitochondrial genes, as well as genes that were not detected in more than 1 cell were discarded. We used markers for Kupffer (*Clec4f, Csf1r*, *C1qc*, *C1qa*, *C1qb*), endothelial (*Kdr*, *Egfl7*, *Igfbp7*, *Aqp1*) and hepatocyte (*Apoa1*, *Apob*, *Pck1*, *G6pc*, *Ttr*) cells for cell identification (Halpern et al., 2017). In order to focus on hepatocytes, we kept only those cells that had a summed expression of hepatocyte markers greater than both the summed expression of Kupffer as well as endothelial cells markers. We also eliminated cells that had less than 0.5% of *Alb* reads. The normalization of the UMI counts was performed with the SCTransform from the Seurat Package, with the regression variable on the condition (uninfected and 2 dpi) (Hafemeister and Satija, 2019). We further ran PCA on the normalized counts and the 3.000 most variable genes. Based on the Jack Straw method we selected the first 20 principal components explaining most of the variability and proceeded with a UMAP low-dimensional projection. Differential analysis between the 2 conditions was performed using the FindMarkers function in Seurat, based on the Wilcox test with a Bonferroni correction. Genes were considered as differentially expressed if they had an absolute average log2 fold-change greater than 0.2 and an adjusted p value smaller than 0.05.

Principal component analysis

Principal component analysis (PCA) was performed on the gene set with a minimum average expression level across conditions of 5 FPKM. Only the 10% most variable (computed on the coefficient of variance) genes were considered for the PCA analysis.

Hierarchical clustering

Hierarchical clustering of different gene sets FPKM or CPM expression as well as metabolite abundance values were performed using a Pearson distance measure with an average clustering method. A k-means ++ (z-norm) clustering approach using ExpressCluster software v1.3 (http://cbdm.hms.harvard.edu/LabMembersPges/SD.html) was performed on the union of differentially modulated genes or metabolites.

Interaction model

For the interaction model (2x2 factorial design) of naive versus LCMV-infected $Ifnar1^{\Delta/\Delta}$ and $Ifnar1^{+/+}$, we quantified gene expression as the number of reads covering each gene. The gene expression on the mouse Ensembl e87 transcripts was quantified from the previously TopHat2 mapped reads with featureCounts (Liao et al., 2014). Raw read counts were normalized with the voom (Law et al., 2014) function of the limma package (Smyth, 2004). Normalized expression values, reported as log2 counts per million (CPM), were then processed through limma's empirical Bayes models. We implemented limma's interaction model as a two ($Ifnar1^{+/+}$, $Ifnar1^{\Delta/\Delta}$) by two (naive, LCMV-infected) factorial design. Genes differentially modulated in the interaction model have been selected based on a minimum log2 CPM of 0, an adjusted p value $\leq$ 0.05 and a minimum log2 fold-change absolute value of 1 (heatmap) or 0.6 (circos plot).

Enrichments and pathway analyses

Enrichment analyses on the union of differential modulated entities (transcripts and/or proteins) specific clusters were done in Cytoscape ClueGO (Bindea et al., 2009) v2.3.3, based on GO (Biological Processes, Molecular Functions, Immune System Process), InterPro, KEGG, Reactome and Wiki Pathways. Terms were called enriched based on a maximum p value of 0.05 and a minimum of 2% gene overlap. GO Term Fusion and grouping was applied. Enriched groups where further ranked according to the group Bonferroni step-down adjusted p value.

For metabolic pathway enrichment analyses, we took the union of differentially expressed genes (expression $\geq$ 1 FPKM, absolute log2 fold-change $\geq$ 0.7, adjusted p value $\leq$ 0.05) and proteins (absolute log2 fold-change $\geq$ 0.25 and adjusted ratio p value $<$ 0.05 and sample p value $<$ 0.05) across all time points. We extracted metabolism-associated genes from KEGG (Kanehisa and Goto, 2000) metabolic pathways database. Only the pathways with q value of enrichment $<$ 0.05 were considered. The enriched pathways are

represented as a circos plot (Krzywinski et al., 2009), with the width of each ribbon in a given category representing the percentage of genes at each time point among all genes leading to enrichments in the respective category. The color gradient (from lighter to darker) represents the percentage of pathways in each category that were found as enriched at a specific time point.

Mass spectrometry data processing

The acquired raw MS data files were processed with msconvert (ProteoWizard Library v2.1.2708) and converted into MASCOT generic format (mgf) files. Peptides were identified by searching the resultant peak lists against the SwissProt mouse database version v2013.01_20130110 (24615 sequences; 14280050 residues) with the search engines MASCOT (v2.3.02, MatrixScience, London, UK) and Phenyx (v2.5.14, GeneBio, Geneva, Switzerland). Submission to the search engines was done via a Perl script that performs an initial search with relatively broad mass tolerances (MASCOT only) on both the precursor and fragment ions (±10 ppm and ± 0.6 Da, respectively). High-confidence peptide identifications were used to recalibrate all precursors and fragment ion masses prior to a second search with narrower mass tolerances (±4 ppm and ± 0.025 Da). Trypsin was chosen as cleavage specificity with the maximum of 1 miscleavage site allowed. Carbamidomethyl cysteine, N-terminal and lysine-modified TMT 6-plex were set as fixed modifications, whereas oxidized methionine was set as a variable modification.

To validate the proteins, MASCOT and Phenyx output files were processed by internally developed parsers. Proteins with ≥ 2 unique peptides above a score T1, or with a single peptide above a score T2, were selected as unambiguous identifications. Additional peptides for these validated proteins with score > T3 were also accepted. For MASCOT and Phenyx, T1, T2, and T3 peptide scores were equal to 16, 40, 10 and 5.5, 9.5, 3.5, respectively (P value $< 10^{-3}$). The validated proteins retrieved by the two algorithms were merged, any spectral conflicts discarded, and grouped according to shared peptides. A false positive detection rate (FDR) of < 1% and < 1% was determined for proteins and peptides, respectively, by applying the same procedure against a reversed database.

The log2 fold-change cutoffs for differential protein modulation were determined based on pairwise comparisons of protein abundances between the two replicates of uninfected control samples across the 3 independent runs. The 2.5% and 97.5% quartiles of the inter-replica pairwise log2 fold-change was computed. Based on the quartile values, we have set the cutoff at 0.25 and −0.25 for up- and down-modulated proteins, respectively. Additional to the log2 fold-change, statistical significance of observed changes was calculated by Isobar software (Breitwieser et al., 2011). Adjusted P value ratio as well as the P value samples as calculated by Isobar were asked to be less than 0.05.

Targeted LC-MS based metabolite quantification

Metabolite measurements using the AbsoluteIDQ p180 Kit (Biocrates Life Science AG) were validated with the supplied software MetIDQ, Version 5-4-8-DB100-Boron-2607 (Biocrates Life Sciences). All metabolite abundances inferior to the limit of detection (LOD) were replaced with a value equal to half the LOD. We eliminated all metabolites that did not have an average abundance superior to LOD in at least one condition. Modulation of metabolites was assessed through a t test between conditions. The cutoffs for the modulation were obtained based on all the inter-replica pairwise comparisons of wild-type uninfected samples. The 2.5 and 97.5 quartiles of these inter-replica log fold-change were used to define the cutoffs. The cutoffs were −0.44 and 0.65 for the whole liver dynamics serum metabolite measurements. For serum measurements of $Ifnar1^{\Delta/\Delta}$ and $Ifnar1^{+/+}$ mice at 1.5 days post infection, based on the same approach, we have set the cutoffs at −0.73 and 0.4. Significance was inferred based on a p value inferior to 0.05. For the interaction model (2x2 factorial design) of naive versus LCMV-infected $Ifnar1^{\Delta/\Delta}$ and $Ifnar1^{+/+}$, we implemented limma's interaction model as a two ($Ifnar1^{+/+}$, $Ifnar1^{\Delta/\Delta}$) by two (naive, LCMV-infected) factorial design. Significantly modulated metabolites in the interaction model were identified by an absolute log2 fold change of 0.7 and a p value inferior to 0.05.

Metabolite tracing quantification

Metabolite tracing data were processed using the TraceFinder 4.1 software (Thermo Scientific).

Immunohistochemistry quantification

Images were photographed using an Olympus BX 53 microscope, and were quantified using HistoQuest TM software (TissueGnostics GmbH, Vienna, Austria).

Statistical information

Data are presented as arithmetic mean ± SEM. Statistical significances were calculated using a Student's t test when comparing two groups or using two-way ANOVA with Bonferroni correction when comparing longitudinal changes. * p < 0.05 ** p < 0.01 *** p < 0.001.

DATA AND CODE AVAILABILITY

Proteomic data (PRIDE: PXD011122) and transcriptomic data (GEO: GSE123688, which includes GEO: GSE118703, GSE123684, GSE118819 and GSE137082 and PRIDE: PXD011122) are deposited in the PRIDE and GEO databases, respectively.

Immunity, Volume *51*

Supplemental Information

Type I Interferon Signaling Disrupts the Hepatic Urea Cycle and Alters Systemic Metabolism to Suppress T Cell Function

Alexander Lercher, Anannya Bhattacharya, Alexandra M. Popa, Michael Caldera, Moritz F. Schlapansky, Hatoon Baazim, Benedikt Agerer, Bettina Gürtl, Lindsay Kosack, Peter Májek, Julia S. Brunner, Dijana Vitko, Theresa Pinter, Jakob-Wendelin Genger, Anna Orlova, Natalia Pikor, Daniela Reil, Maria Ozsvár-Kozma, Ulrich Kalinke, Burkhard Ludewig, Richard Moriggl, Keiryn L. Bennett, Jörg Menche, Paul N. Cheng, Gernot Schabbauer, Michael Trauner, Kristaps Klavins, and Andreas Bergthaler

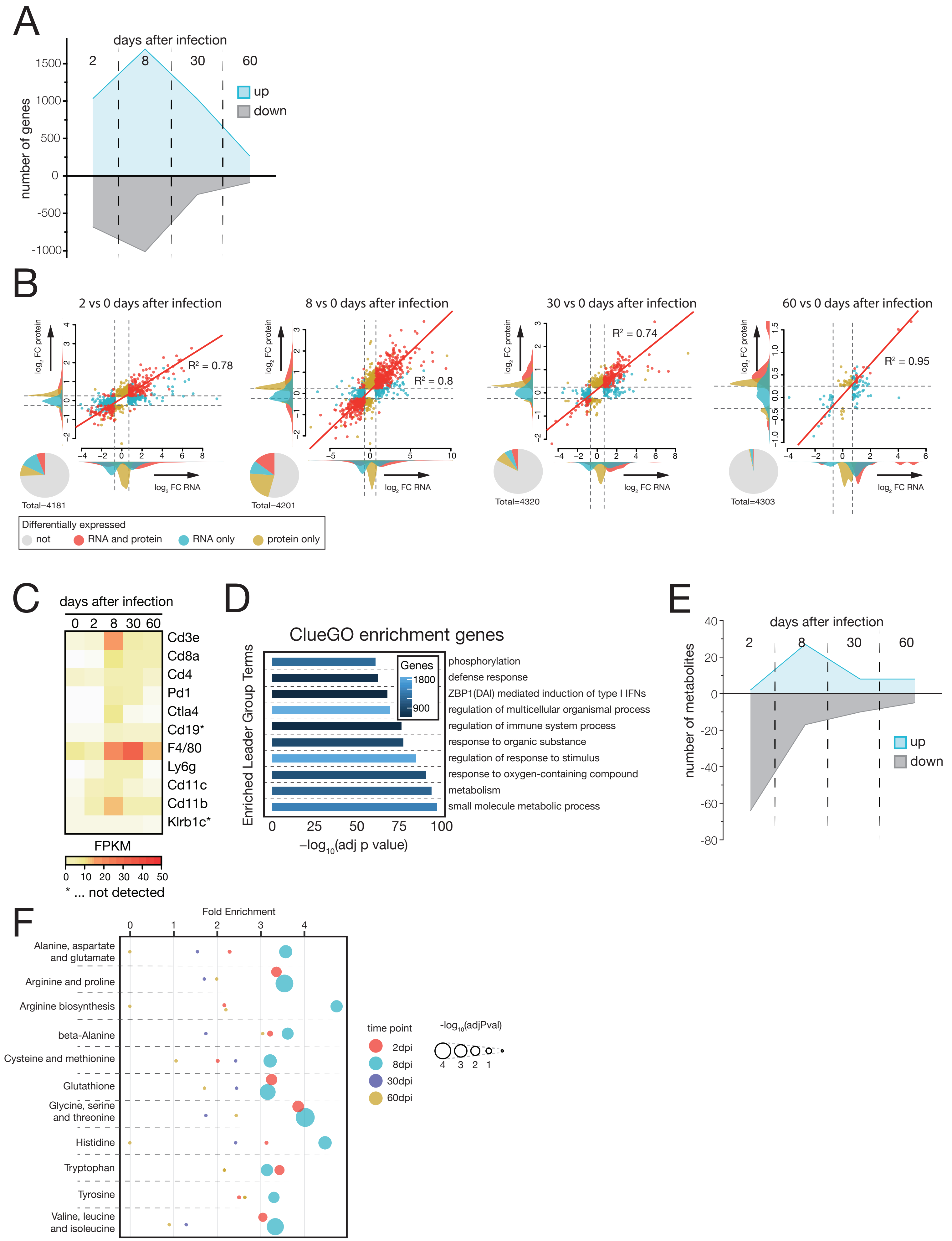
A
days after infection
2
8
30
60
number of genes
1500
1000
500
0
-500
-1000
up
down
B
2 vs 0 days after infection
8 vs 0 days after infection
30 vs 0 days after infection
60 vs 0 days after infection
log2 FC protein
log2 FC RNA
R² = 0.78
R² = 0.8
R² = 0.74
R² = 0.95
Total=4181
Total=4201
Total=4320
Total=4303
Differentially expressed
not
RNA and protein
RNA only
protein only
C
days after infection
0 2 8 30 60
Cd3e
Cd8a
Cd4
Pd1
Ctla4
Cd19*
F4/80
Ly6g
Cd11c
Cd11b
Klrb1c*
FPKM
0 10 20 30 40 50
* ... not detected
D
ClueGO enrichment genes
Enriched Leader Group Terms
Genes
1800
900
phosphorylation
defense response
ZBP1(DAI) mediated induction of type I IFNs
regulation of multicellular organismal process
regulation of immune system process
response to organic substance
regulation of response to stimulus
response to oxygen-containing compound
metabolism
small molecule metabolic process
0 25 50 75 100
–log10(adj p value)
E
days after infection
2
8
30
60
number of metabolites
40
20
0
-20
-40
-60
-80
up
down
F
Fold Enrichment
0 1 2 3 4
Alanine, aspartate and glutamate
Arginine and proline
Arginine biosynthesis
beta-Alanine
Cysteine and methionine
Glutathione
Glycine, serine and threonine
Histidine
Tryptophan
Tyrosine
Valine, leucine and isoleucine
time point
2dpi
8dpi
30dpi
60dpi
-log10(adjPval)
4 3 2 1

Figure S1. Systems biology approach to transcriptomic, proteomic and metabolomic changes in liver tissue and systemic metabolism during LCMV Cl13 infection. Related to Figure 1.

(A) Number of significantly up- or downregulated genes in LCMV-infected liver tissue 2, 8, 30 or 60 days after infection compared to naïve liver tissue (n = 3). (B) Correlation of transcriptomic and proteomic changes at the corresponding time points after LCVM infection (n = 3). (C) Heatmap of expression values (FPKM) of indicated immune cell markers in liver tissue during the course of LCMV infection (n = 3). (D) Enriched GO terms and pathways (ClueGO) on the union of significantly differentially expressed transcripts at any time point (n = 3). (E) Number of significantly up- or downregulated serum metabolites of LCMV-infected wild type mice compared to naïve serum metabolite levels (n = 4). (F) Enrichment analyses of significantly regulated transcripts for amino acid metabolic pathways (KEGG) at the indicated time points after LCMV infection (n = 3). For (A-F) transcriptomic and proteomic data is derived from one experiment.

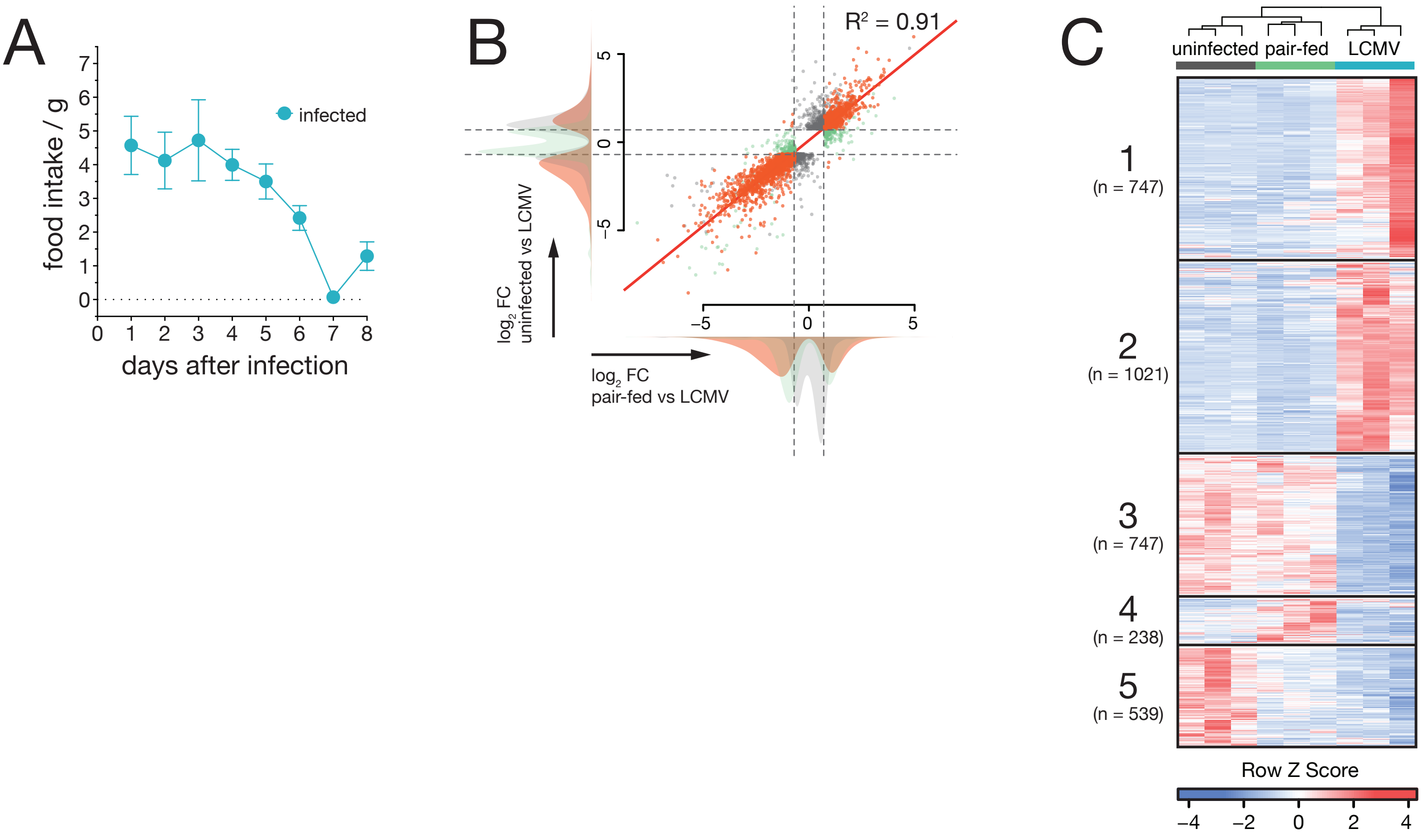

Figure S2. Transcriptome analyses of naïve and pair-fed compared to LCMV-infected animals. Related to Figure 2.

(A) Food intake of LCMV-infected animals (n = 3) up to 8 days post infection. (B) Correlation of significantly regulated transcripts in the livers of naïve and pair-fed animals (8 days) compared to LCMV-infected animals (n = 3). (C) Hierarchical clustering (FPKM, k-means, Pearson's correlation) of significantly deregulated transcripts in liver tissue of naïve, pair-fed and LCMV-infected animals (n = 3). For (A-C) transcriptomic data is derived from one experiment. Symbols represent the arithmetic mean ±S.E.M.

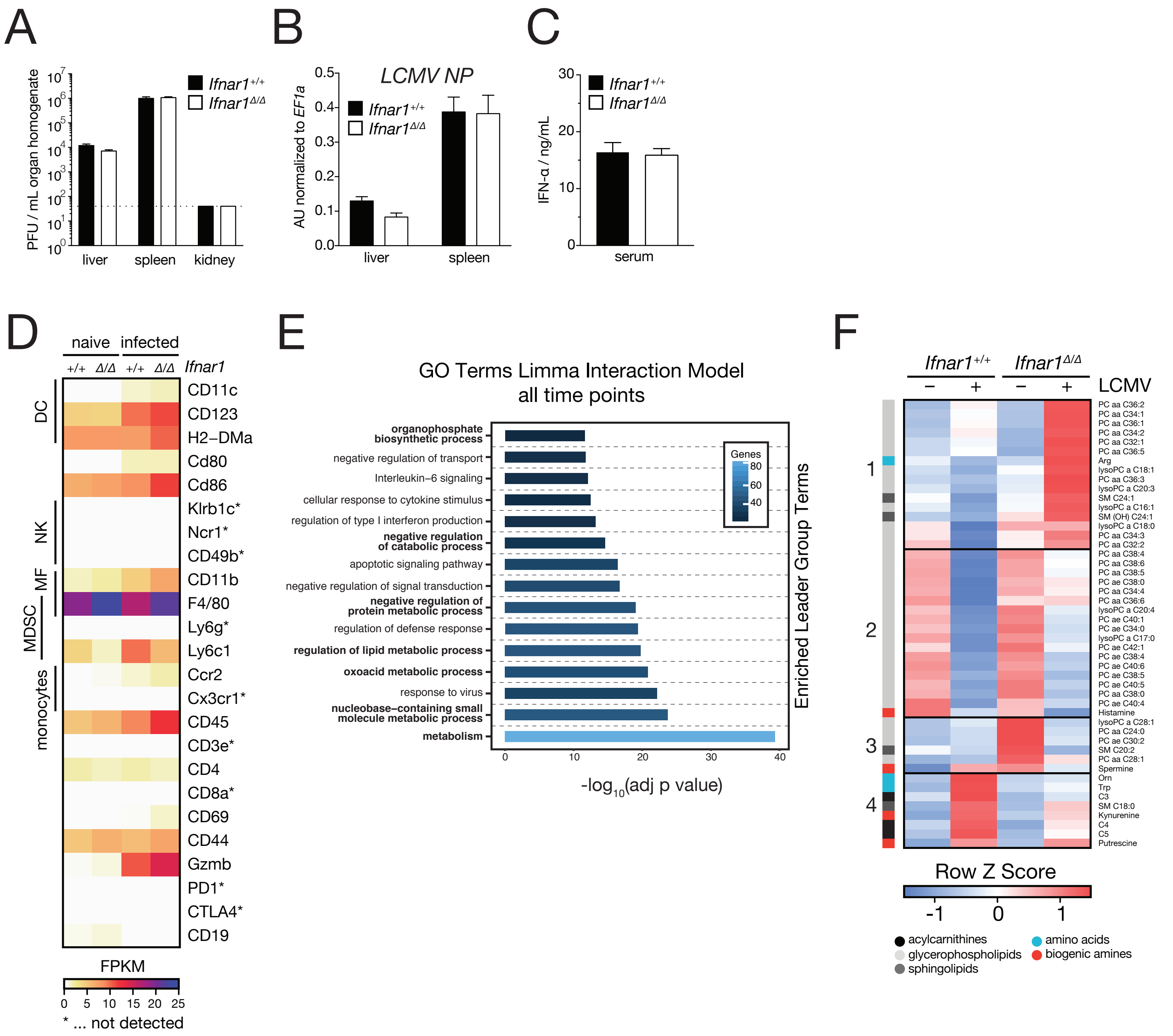

Figure S3. Transcriptome and systemic metabolome analyses of LCMV Cl13 infected *Alb-Cre ERT2* $Ifnar1^{fl/fl}$ mice. Related to Figure 3.

(A) Viremia and (B) RNemia in organs (n = 4), and (C) IFN-α serum levels of *Alb-Cre ERT2* $Ifnar1^{fl/fl}$ ($Ifnar1^{\Delta/\Delta}$) and $Ifnar1^{+/+}$ mice 1.5 days after infection (n = 4). (D) Heatmap of expression values (FPKM) of the indicated immune cell markers in naïve or infected $Ifnar1^{+/+}$ and $Ifnar1^{\Delta/\Delta}$ mice (n = 3). (E) Enriched GO terms on the union of significantly regulated (limma interaction model) genes (n = 3). (F) Significantly regulated serum metabolites in naïve and infected $Ifnar1^{\Delta/\Delta}$ and $Ifnar1^{+/+}$ animals (n = 3, k-means, Pearson's correlation). For (A-C) one of two representative experiments are shown. For (D-F) transcriptomic and metabolomic data is derived from one experiment. AU = arbitrary units. Symbols represent the arithmetic mean ±S.E.M. Dotted line implicates limit of detection. ns = not significant * $P < 0.05$ ** $P < 0.01$ *** $P < 0.001$(Student's t-test).

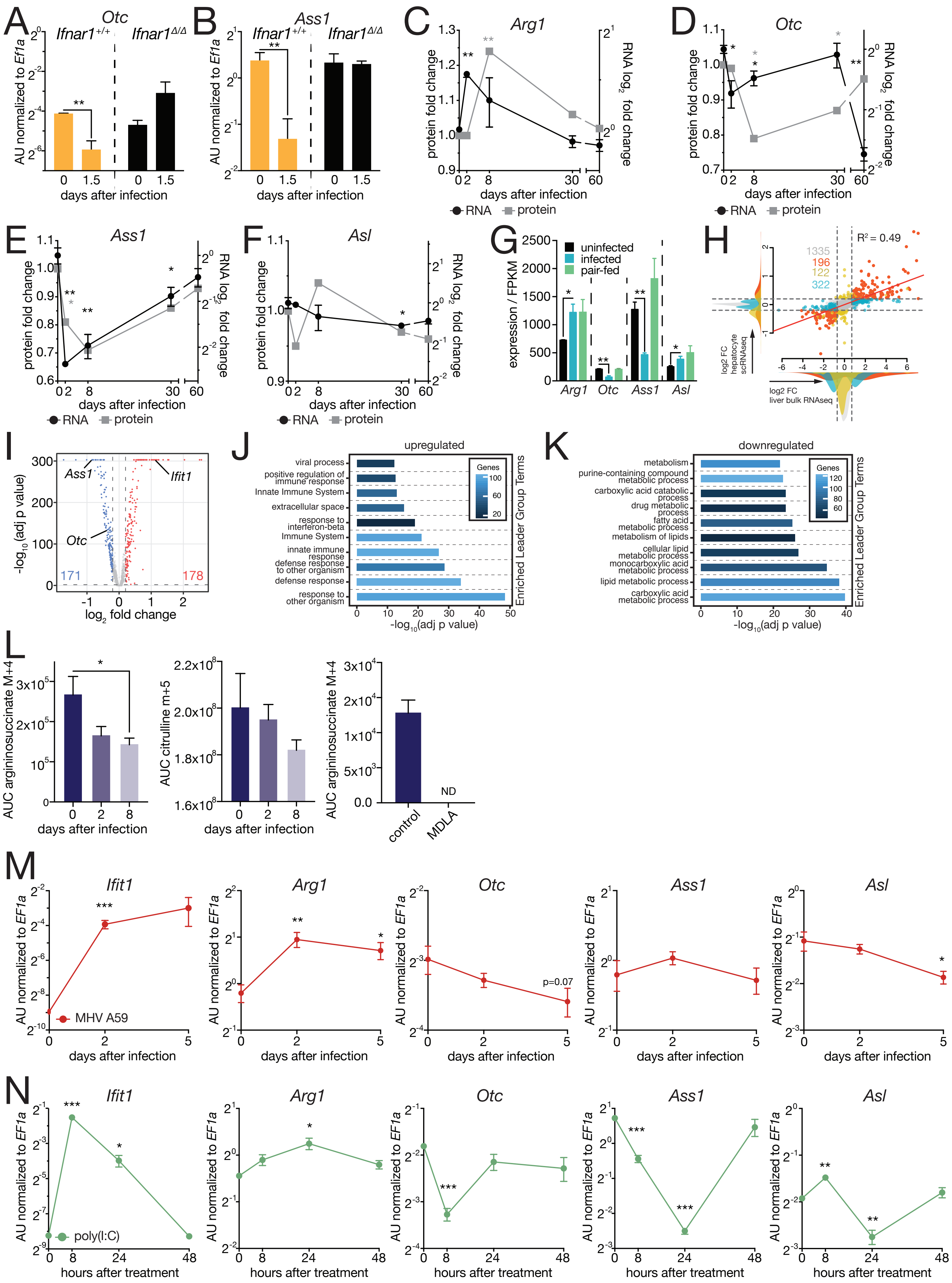
A
Otc
Ifnar1+/+
Ifnar1Δ/Δ
AU normalized to Ef1a
days after infection
B
Ass1
C
Arg1
protein fold change
RNA log2 fold change
days after infection
RNA
protein
D
Otc
E
Ass1
F
Asl
G
uninfected
infected
pair-fed
expression / FPKM
Arg1
Otc
Ass1
Asl
H
R² = 0.49
1335
196
122
322
log2 FC hepatocyte scRNAseq
log2 FC liver bulk RNAseq
I
-log10(adj p value)
log2 fold change
Ass1
Ifit1
Otc
171
178
J
upregulated
viral process
positive regulation of immune response
Innate Immune System
extracellular space
response to interferon-beta
Immune System
innate immune response
defense response to other organism
defense response
response to other organism
Genes
Enriched Leader Group Terms
-log10(adj p value)
K
downregulated
metabolism
purine-containing compound metabolic process
carboxylic acid catabolic process
drug metabolic process
fatty acid metabolic process
metabolism of lipids
cellular lipid metabolic process
monocarboxylic acid metabolic process
lipid metabolic process
carboxylic acid metabolic process
L
AUC argininosuccinate M+4
AUC citrulline m+5
days after infection
ND
control
MDLA
M
Ifit1
Arg1
Otc
Ass1
Asl
MHV A59
p=0.07
N
poly(I:C)
hours after treatment

Figure S4. IFNAR1 signaling and viral infection perturb the urea cycle in hepatocytes. Related to Figure 4.

Real-time PCR of *Otc* (A) and *Ass1* (B) in *Alb-Cre ERT2 Ifnar1fl/fl* (*Ifnar1*$^{\Delta/\Delta}$) and *Ifnar1*$^{+/+}$ mice 1.5 days after infection (n = 3-4). (D-F) Long-term kinetics of urea cycle gene transcript and protein abundances of *Arg1*, *Otc*, *Ass1* and *Asl* in liver tissue upon LCMV-infection (n = 3). (G) Expression values (FPKM) of *Arg1*, *Otc*, *Ass1* and *Asl* of naïve, pair-fed and LCMV-infected (8 days after infection) wild type animals (n = 3). (H) Correlation plot (Spearman correlation) of significantly changed genes in scRNA-seq dataset (n = 2, pooled for each condition) compared to RNA-seq data obtained from bulk liver tissue (n = 3, Figure 1). (I) Volcano plot of scRNA-seq data displaying up- and downregulated transcripts (n = 2, pooled for each condition); arrows indicate the urea cycle genes *Otc* and *Ass1* and the type I interferon stimulated gene *Ifit1*. (J, K) Enriched GO terms of significantly up- and downregulated genes in hepatocytes derived from naïve or LCMV-infected mice (2 days, n = 2, pooled for each condition). (L) Detection of ^{13}C labeled reaction products for ASS1 ($^{13}C_4$ argininosuccinate) and OTC ($^{13}C_5$ citrulline) after pulsing liver lysates of naïve or LCMV-infected animals (2 and 8 days after infection) for 30 minutes with the respective ^{13}C labeled substrates for OTC and ASS1 (n = 4-5) and detection of the ASS1 reaction product $^{13}C_4$ argininosuccinate in naïve liver lysates in the presence or absence of the ASS1 inhibitor α-methyl-DL-aspartic acid (n = 3). (M) Expression of *Ifit1* and urea cycle-associated genes in liver tissue of mice infected with murine hepatitis virus (MHV) strain A59 measured by real-time PCR (n = 5). (N) Expression of *Ifit1* and urea cycle-associated genes in liver tissue upon poly(I:C) treatment of wild type mice measured by real-time PCR (n = 3). For (A-B) one of two representative experiments are shown. For (D-N) data is derived from one experiment. AU = arbitrary units, AUC = area under curve. Symbols represent the arithmetic mean ±S.E.M. ns = not significant * $P < 0.05$ ** $P < 0.01$ (Student's t-test).

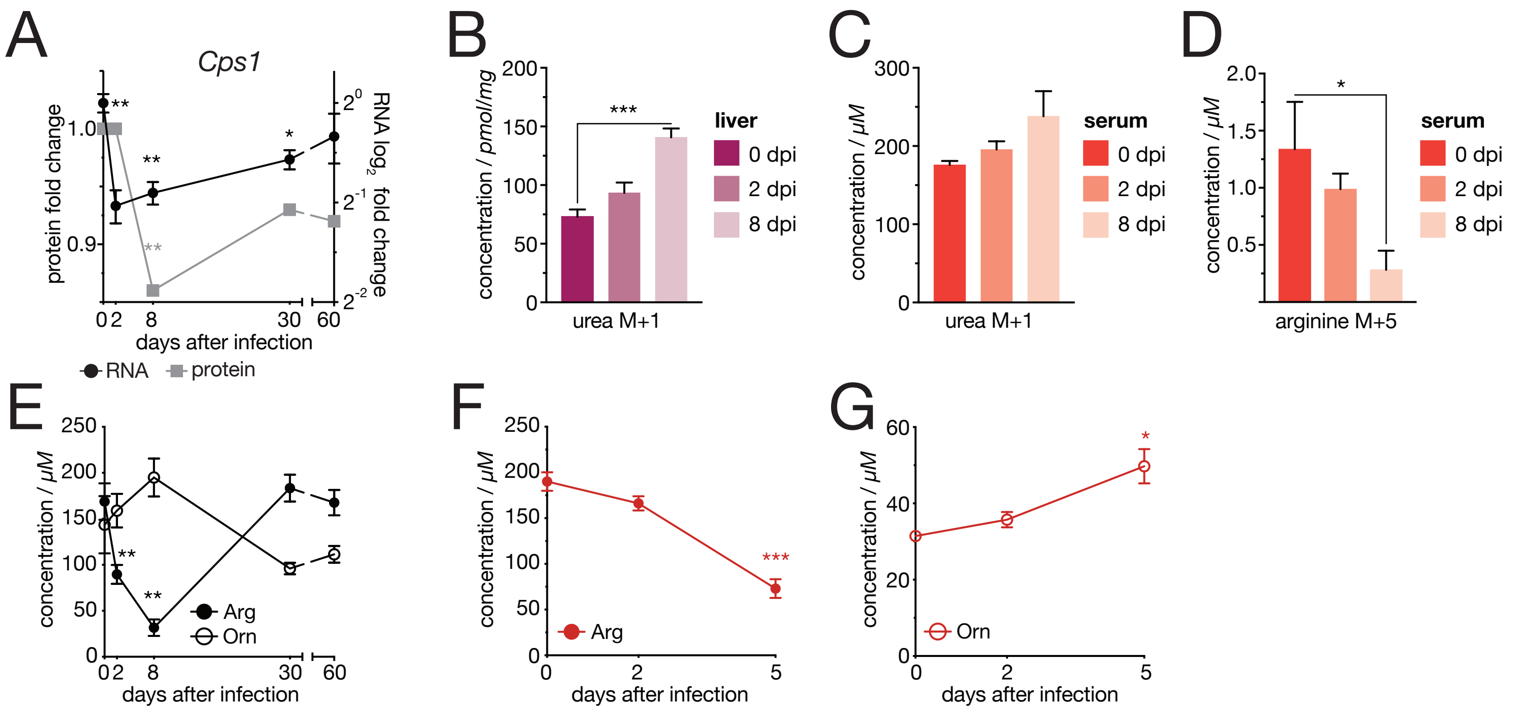

Figure S5. Viral infection stalls the hepatic urea cycle and correlates with changes in serum levels of arginine and ornithine. Related to Figure 5.

(A) Transcript and protein levels of *Cps1* in liver tissue of mice infected with LCMV up to 60 days after infection (n = 3). Concentrations of $^{13}C_1$ labeled urea in (B) liver tissue, (C) serum and (D) $^{13}C_5$ labelled arginine in serum of naïve and LCMV-infected (2 and 8 days after infection) wild type mice (n= 3-6). (E) Serum levels of arginine and ornithine in LCMV-infected wild type mice up to 60 days after infection. (F, G) Serum levels of arginine and ornithine in mice infected with murine hepatitis virus (MHV) strain A59 up to 5 days after infection (n = 5). For (E) two individual biological experiments were pooled. For (A–D and F-G) transcriptomic, metabolomic and metabolite tracing data is derived from one experiment. Symbols represent the arithmetic mean ±S.E.M. ns = not significant * P < 0.05 ** P < 0.01 (Student's t-test).

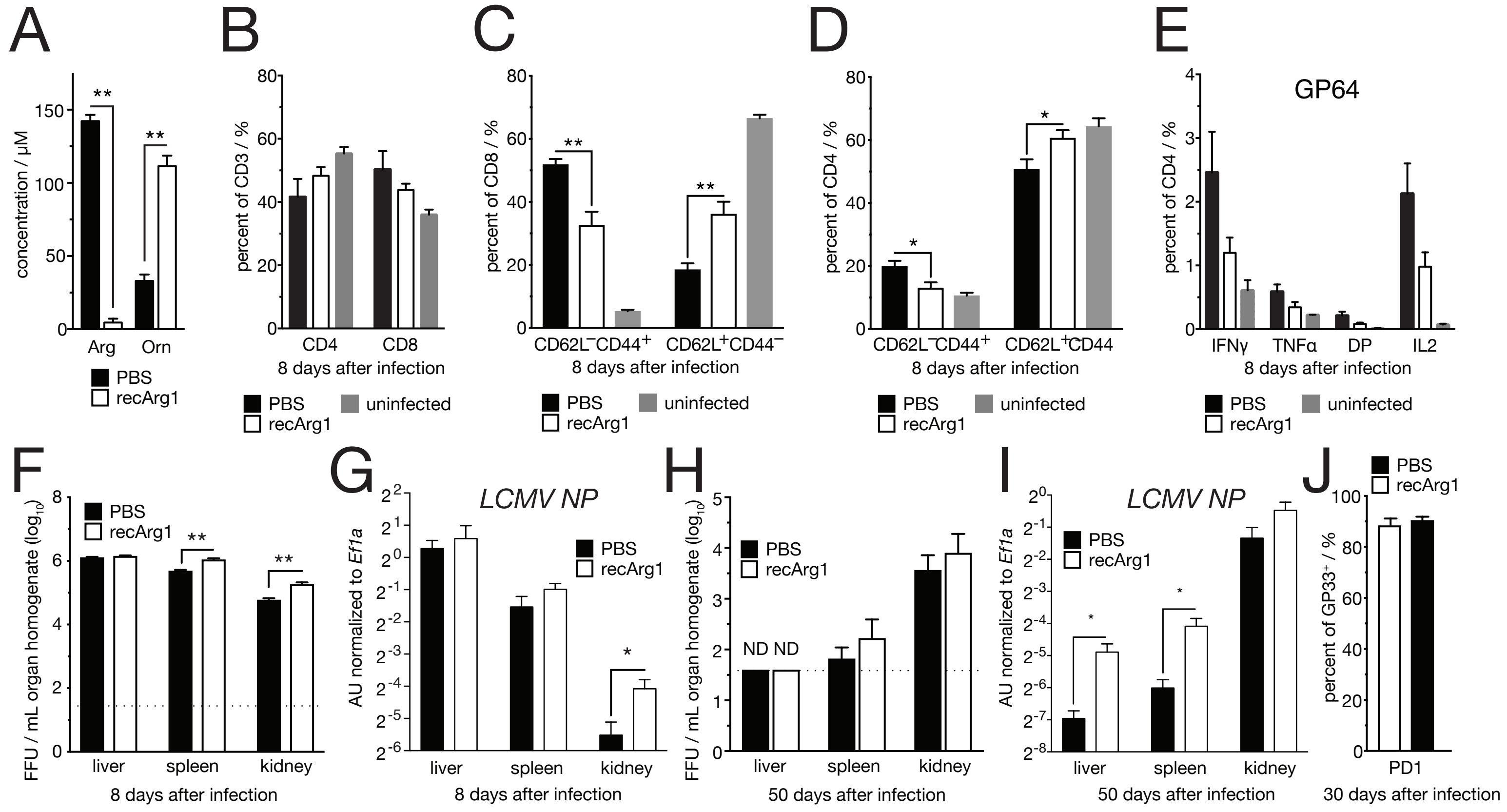

Figure S6. Arginase1 treatment does not affect splenic CD4 and CD8 T cell abundances and mildly impairs antiviral CD4 T cell responses and affects organ viral load. Related to Figure 6.

(A) Serum arginine and ornithine levels in wild type mice (n = 4) 2 days after treatment with pegylated recombinant human Arginase 1 (recArg1, BCT-100). (B) Percentage of $CD4^+$ and $CD8^+$ cells of $CD3^+$ splenocytes at 8 days after LCMV clone 13 infection and recArg1 treatment (n = 5). (C) $CD62L^-CD44^+$ (effector) and $CD62L^+CD44^-$ (naïve) CD8 and (D) CD4 splenic T cells (n = 5). (E) IFNγ, TNFα and IL-2 production of LCMV GP64-specific splenic CD4 T cells 8 days after infection. (F) Viraemia and (G) RNemia of liver, spleen and kidney 8 days after infection (n = 5). (H) Viraemia and (I) RNemia of liver, spleen and kidney 50 days after infection (n = 4-5). (J) PD1 expression on GP33-specific CD8 T cells in blood 30 days after infection (n = 5). For (B-I) one of at least two representative experiments are shown. For (A) data is derived from one experiment. AU = arbitrary units. Symbols represent the arithmetic mean ±S.E.M. n = 4-5. ns = not significant. ND = not detected. Dotted line implicates limit of detection. * $P < 0.05$ ** $P < 0.01$ (Student's t-test).

Table S1: Transcriptomic and proteomic data of LCMV-infected wild type mice. Related to Figure 1.

Table S2: Enrichment cluster analysis of differentially expressed genes. Related to Figure 1.

Table S3: Metabolomics data of LCMV-infected mice. Related to Figure 1.

Table S4: Transcriptomic and gene ontology enrichment data of pair-feeding experiment. Related to Figure 2.

Table S5: Transcriptomic and gene ontology enrichment data of *Alb-Cre ERT2 Ifnar1* mice. Related to Figure 3.
